# Evx1 and Evx2 specify excitatory neurotransmitter fates and suppress inhibitory fates through a Pax2-independent mechanism

**DOI:** 10.1186/s13064-016-0059-9

**Published:** 2016-02-19

**Authors:** José L. Juárez-Morales, Claus J. Schulte, Sofia A. Pezoa, Grace K. Vallejo, William C. Hilinski, Samantha J. England, Sarah de Jager, Katharine E. Lewis

**Affiliations:** Department of Biology, Syracuse University, 107 College Place, Syracuse, NY 13244 USA; Department of Physiology, Development and Neuroscience, University of Cambridge, Downing Street, Cambridge, CB2 3DY UK; Department of Neuroscience and Physiology, SUNY Upstate Medical University, 505 Irving Avenue, Syracuse, NY 13210 USA

**Keywords:** Spinal cord, Interneuron, Zebrafish, Evx, Pax2, Glutamatergic, Neurotransmitter, CNS, Transcription factor, V0

## Abstract

**Background:**

For neurons to function correctly in neuronal circuitry they must utilize appropriate neurotransmitters. However, even though neurotransmitter specificity is one of the most important and defining properties of a neuron we still do not fully understand how neurotransmitter fates are specified during development. Most neuronal properties are determined by the transcription factors that neurons express as they start to differentiate. While we know a few transcription factors that specify the neurotransmitter fates of particular neurons, there are still many spinal neurons for which the transcription factors specifying this critical phenotype are unknown. Strikingly, all of the transcription factors that have been identified so far as specifying inhibitory fates in the spinal cord act through Pax2. Even Tlx1 and Tlx3, which specify the excitatory fates of dI3 and dI5 spinal neurons work at least in part by down-regulating Pax2.

**Methods:**

In this paper we use single and double mutant zebrafish embryos to identify the spinal cord functions of Evx1 and Evx2.

**Results:**

We demonstrate that Evx1 and Evx2 are expressed by spinal cord V0v cells and we show that these cells develop into excitatory (glutamatergic) Commissural Ascending (CoSA) interneurons. In the absence of both Evx1 and Evx2, V0v cells still form and develop a CoSA morphology. However, they lose their excitatory fate and instead express markers of a glycinergic fate. Interestingly, they do not express Pax2, suggesting that they are acquiring their inhibitory fate through a novel Pax2-independent mechanism.

**Conclusions:**

Evx1 and Evx2 are required, partially redundantly, for spinal cord V0v cells to become excitatory (glutamatergic) interneurons. These results significantly increase our understanding of the mechanisms of neuronal specification and the genetic networks involved in these processes.

**Electronic supplementary material:**

The online version of this article (doi:10.1186/s13064-016-0059-9) contains supplementary material, which is available to authorized users.

## Background

Hundreds of millions of people across the world are affected by neurological diseases and injuries. Understanding how functional neuronal circuitry is established in the vertebrate central nervous system (CNS) is essential for developing better treatments for these conditions. How neuronal circuitry develops is also a fundamental question in developmental neuroscience. To answer this question, we need to identify how the functional properties of distinct neurons are specified; since these properties determine which circuits the neurons participate in, the functional roles that the neurons have within those circuits and the resulting outputs of the circuitry. The spinal cord is a powerful system for establishing fundamental principles of neuronal fate specification, function and circuit assembly, as it is relatively simple and experimentally tractable compared to the brain. This has enabled considerable progress in establishing the functions of different ventral spinal cord interneurons in locomotor circuitry (e.g. [1–9]). However, we still know relatively little about how the functional properties of these cells are determined.

For neurons to function correctly they must synthesize and utilize correct neurotransmitters. Within neuronal circuitry, if they inhibit rather than excite their synaptic partners, or vice versa, then the behaviors and functional outputs of those circuits will be dramatically disturbed, and may give rise to pathological conditions. For example, disruptions in the balance of excitatory and inhibitory neurons in the CNS have been implicated in epilepsy, autism, Alzheimer’s and many other neurological disorders (e.g. [10–13]). However, even though neurotransmitter specificity is one of the most important and defining properties of a neuron we still do not fully understand how neurotransmitter fates are specified during development.

Many neuronal properties are determined by the transcription factors that cells express as they start to differentiate. We already know a few transcription factors (e.g. Ptf1a, Lhx1, Lhx5, Lbx1, Pax2) that specify the inhibitory (GABAergic and/or glycinergic) fates of several subsets of spinal interneurons [14–18]. Strikingly, most of these transcription factors function in dorsal spinal neurons and all of them act through Pax2 [14–21]. In contrast, we only know two transcription factors, Tlx1 and Tlx3, that are required for the specification of excitatory (glutamatergic) fates and these are only expressed in dorsal dI3, dI5 and DIL_B_ cells [15, 16, 22]. Interestingly, Tlx1 and Tlx3 determine the glutamatergic fates of dI3 and dI5 cells at least in part by down-regulating Pax2 [15]. These results suggest that Pax2 is a crucial player in neurotransmitter fate specification with its presence being required for inhibitory fates and its absence required for excitatory fates. However, we still do not know which transcription factors regulate the neurotransmitter fates of many excitatory spinal neurons, including those in the ventral spinal cord, whose correct functional specification is essential for locomotion.

In this paper we identify two transcription factors, Evx1 and Evx2, which are required for a subset of excitatory fates in the ventral spinal cord. In mammals, the spinal cord expression of Evx1 and Evx2 is restricted to a population of cells located in an intermediate dorso-ventral position corresponding to V0 cells (e.g. [23–28]). V0 cells are post-mitotic cells that form from the p0 (Dbx1-positive, Nkx6.2-negative) progenitor domain [23, 27–29]. These cells develop into interneurons that are important components of locomotor circuitry and they can be subdivided into an Evx1-positive sub-population called V0_v_ cells and an Evx1-negative sub-population called V0_D_ cells. These names reflect the fact that V0v cells form more ventrally than V0_D_ cells (e.g. [23–28, 30–34]). Evx2 is expressed in a similar pattern to Evx1 in the mouse CNS, suggesting that it may also be expressed by V0v cells. This is consistent with the observation that Evx2 spinal cord expression is lost in mouse *Evx1* mutants [23]. However, co-expression of Evx1 and Evx2 in the mouse spinal cord has not yet been demonstrated [24].

In mammals, both V0v and V0_D_ interneurons are crucial for correct left-right alternation during locomotion, with V0v cells in particular being required for hindlimb left-right alternation during fast locomotion [9, 34]. While the functions of V0 cells in specific behaviors have so far only been assayed in mouse, these cells have highly conserved commissural axon trajectories in all animals examined so far ([23–28, 32, 33, 35, 36]; this paper), suggesting that their functional properties are likely to be highly conserved across the vertebrate lineage. However, when we started this work the neurotransmitter phenotype of V0v cells had not been identified.

In zebrafish, *evx1* and *evx2* are expressed in a similar intermediate dorsal-ventral spinal cord position to that observed in other vertebrates [26, 32, 33], although again, co-expression of these two genes has not previously been demonstrated. In this paper, we confirm that *evx1* and *evx2* are co-expressed by V0v cells and we show that V0v cells are glutamatergic and have a Commissural Ascending (Comissural Secondary Ascending or CoSA) morphology. We also provide the first analysis of *evx1;evx2* double mutants in any vertebrate and the first analysis of the spinal cord phenotype of *evx2* mutants. Significantly, we demonstrate that Evx1 and Evx2 are required, partially redundantly, to specify the glutamatergic fates of V0v cells. Given that we know so little about how excitatory fates are specified in the spinal cord and particularly the ventral spinal cord, these findings add considerably to our understanding of CNS circuit development.

In the absence of both Evx1 and Evx2, V0v cells lose their glutamatergic fates but other functional characteristics like soma/cell body morphology and axon trajectory are unchanged. In addition, and in contrast to a previously described mouse *Evx1* mutant [23], these cells do not express markers of neighboring cell types. This suggests that V0v cells are not transfating into a different class of neuron; they have just changed some of their functional properties. Strikingly, in *evx1;evx2* double mutants V0v cells become inhibitory, but they do not express Pax2, suggesting that they are acquiring their inhibitory fates through a novel Pax2-independent mechanism.

## Methods

### Ethics approval

All zebrafish experiments in this research were approved either by the UK Home Office or by the Syracuse University IACUC committee.

### Zebrafish husbandry and fish lines

Zebrafish (*Danio rerio*) were maintained on a 14 h light/10 h dark cycle at 28.5 °C and embryos were obtained from natural, paired and/or grouped spawnings of wild-type (WT) adults (AB, TL or AB/TL hybrids), identified heterozygous or homozygous *Tg*(*slc17a6:EGFP)* (used to be called *Tg(vGlut2a:EGFP)*; [36, 37]), *Tg(evx1:EGFP)*^*SU1*^ or *Tg(evx1:EGFP)*^*SU2*^ adults, double heterozygous *evx1*^*i232*^*;evx2*^*sa140*^ mutants or double heterozygous *evx1*^*i232*^*;evx2*^*sa140*^ mutants that also carried one of the *Tg(evx1:EGFP)* lines (see below). Embryos were reared at 28.5 °C and staged by hours post fertilization (h), days post or prim staging/or prim staging [38].

**Fig. 1:**
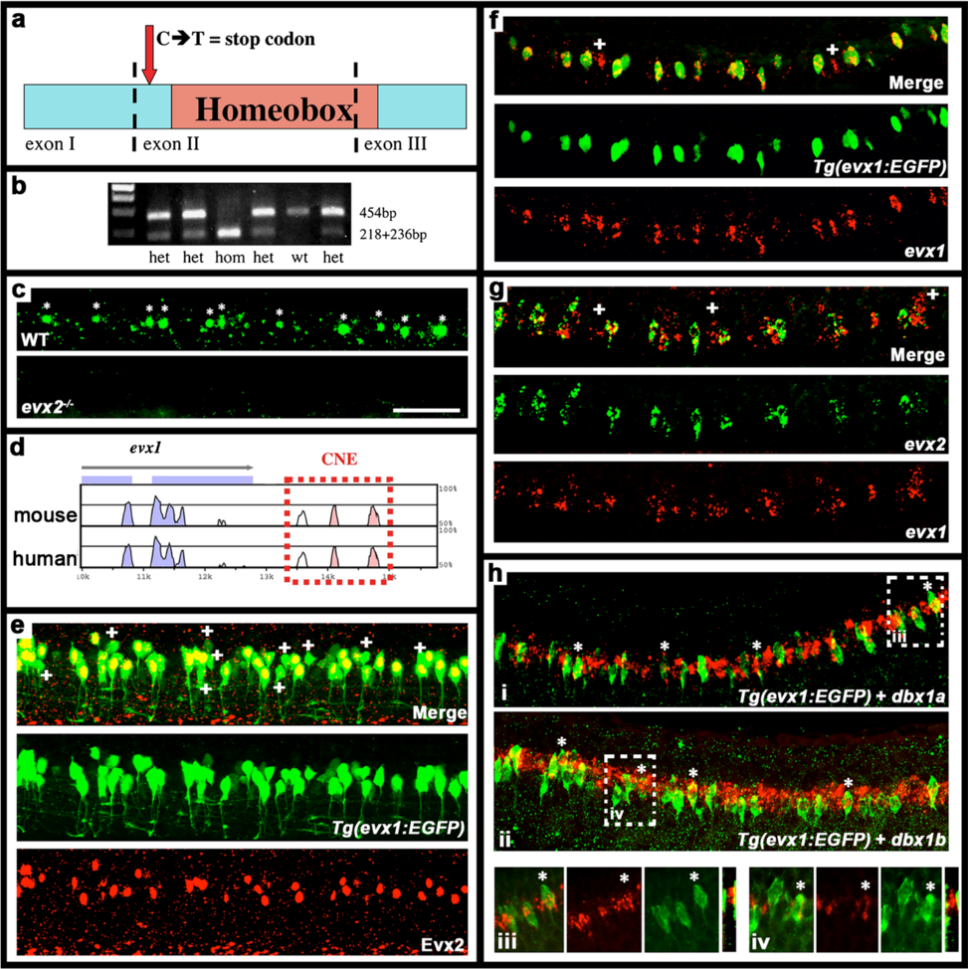
V0v cells co-express *evx1* and *evx2*. **a** Schematic of Evx2 showing exon boundaries (dotted lines), homeobox-domain (pink) and location of *evx2* ^*sa140*^ mutation (red arrow). **b** Examples of genotyping WT, heterozygous and homozygous *evx2* ^*sa140*^ mutant embryos using gel electrophoresis (see methods). The two fragments from the mutant allele restriction product run at the same position on the gel. **c**, **e**-**h** lateral views, dorsal up, anterior left, of spinal cord at 24 h (**f**-**h**) or 27 h (**c **& **e**). **c** Evx2 immunohistochemistry on WT (*top panel*) and homozygous *evx2* ^*sa140*^ mutant (*bottom panel*) embryos. Stars indicate Evx2-expressing cells. Mutant embryos have no Evx2 expression. **d** Schematic showing Shuffle-LAGAN analysis of *evx1* genomic region with zebrafish sequence as baseline compared to orthologous regions in mouse and human genomes. Conserved coding sequences are indicated in blue, arrow indicates 5'-3' orientation. CNEs in 3’ region are indicated in pink. The region amplified to create transgenic lines is indicated with red dotted lines. **e**-**h** Double staining for (**e**) EGFP (green) and Evx2 (red) in *Tg(evx1:EGFP)* ^*SU1*^ embryos, (**f**) EGFP (green) and *evx1* (red) in *Tg(evx1:EGFP)* ^*SU1*^ embryos, (**g**) *evx1* (red) and *evx2* (green) in WT embryos, (**h**) EGFP (green) and *dbx1a* (red) in *Tg(evx1:EGFP)* ^*SU1*^ embryos (i and iii) and EGFP (green) and *dbx1b* (red) in *Tg(evx1:EGFP)* ^*SU1*^ embryos (ii and iv). In **e**-**g** and **h**_iii - **h**_iv, merged and single channel views are provided. White crosses indicate cells that only express *evx1*. In (**f**) these probably represent cells that have just started to express *evx1* as there is a delay in expression of EGFP. White stars in **h** indicate double-labelled cells. Three wider panels at bottom of **h** (iii and iv) are magnified single-confocal-plane views of white dotted rectangle regions in panels **h**_i and **h**_ii respectively. Thin panel on RHS in each case shows a cross-section projection (slice) created in Image J confirming that GFP expression is lateral to *dbx* expression. Scale bar: 50 μm (**c** & **e**-**h**)

The *evx1*^*i232*^ mutation has been described before [39]. The *evx2*^*sa140*^ mutant was received from the Wellcome Trust Sanger Centre, (https://www.sanger.ac.uk/sanger/Zebrafish_Zmpbrowse). Both mutations produce a single base pair change (a C to a T in the case of *evx2*^*sa140*^) that results in a premature stop codon before the homeobox ([39]; Fig. 1a). Therefore, the truncated proteins, if formed, will lack DNA binding domains. The *evx2*^*sa140*^ mutation creates a *BfaI* recognition site that enables us to genotype individual fish and embryos (see below; Fig. 1b). In this paper we demonstrate that the *evx2*^*sa140*^ allele does not make Evx2 protein (Fig. 1c), strongly suggesting that it is a null allele. The *evx1*^*i232*^ mutant is also probably a null allele [39]. However, in contrast to the *evx1*^*i232*^ mutant which is viable, the *evx2*^*sa140*^ mutant is embryonic lethal (see Results and Additional file 1: Results).

### Genotyping

Genotyping of mutant alleles was performed on both live adults and fixed embryos using DNA extracted from fin clips and dissected heads respectively. Fin clipping and *evx1* genotyping were performed as in [39]. To extract DNA from embryos, heads were removed in 80 % glycerol / 20 % PBS with insect pins. Embryonic trunks were stored in 70 % glycerol / 30 % PBS at 4 °C for later analysis. Heads were incubated in 50 μL of Proteinase K buffer solution (1 M Tris-HCl, pH 8.2; 0.5 M EDTA; 1 M NaCl; 20 % SDS; 10 mg/ml Proteinase K) for 2 h at 55 °C. Proteinase K was heat inactivated at 100 °C for 10 min and tubes were centrifuged for 20 min at 13,000 rpm. DNA was precipitated with 100 % ethanol at -20 °C overnight, centrifuged to pellet the DNA and re-suspended in 20 μL of water. 2 μL of DNA was used for each PCR.

The *evx2*^*sa140*^ mutation creates a *BfaI* recognition site. A genomic region flanking the mutation site was PCR amplified using the following conditions: 94 °C for 60 s, followed by 5 cycles of 92 °C for 30 s, 54 °C for 30 s, 72 °C for 60 s; followed by 40 cycles of 92 °C for 20 s, 52 °C for 30 s, and 72 °C for 60 s, followed by a final extension at 72 °C for 5 min. Forward primer: GTAATGCGATCCCAAAACG. Reverse primer: TTATTTTAGATTTGGCAATGG.

PCR products were digested with *BfaI* and analysed on a 1 % agarose gel. The WT product is 454 bp, whereas the mutant product is cut into 218 bp and 236 bp fragments. These fragments are close enough in size that they are usually detected as one band on an agarose electrophoresis gel (Fig. 1b).

### Creation of *Tg(evx1:EGFP)* lines

Potential *evx1* enhancer regions were identified by multispecies sequence comparisons using the global alignment program Shuffle-LAGAN [40] and visualized using VISTA [41]. Zebrafish (*Danio rerio*) *evx1* genomic sequence (ENSDARG00000099365) and orthologous sequences from human (ENSG00000106038) and mouse (ENSMUSG00000005503) were obtained from Ensembl (http://www.ensembl.org). Zebrafish sequence was used as the baseline and annotated using exon/intron information from Ensembl. The alignment was performed using a 100 bp window and a cutoff score of 70 % identity. A multi-species comparison of approximately 23Kb of *Danio rerio* genomic DNA sequence containing *evx1* and extending 5 Kb into flanking regions revealed high conservation in both coding and non-coding sequences among compared species. We identified three Conserved Non-coding Elements (CNEs) located 5’ and 3’ to *evx1*. The first is located 79 bp upstream of zebrafish *evx1* coding sequence and extends over 100 bp. The other two are located 3' to *evx1*. One is 2354 bp downstream of the stop codon and is 184 bp long whereas the other is 2979 bp downstream of the stop codon and extends over 140 bp (Fig. 1d). One amplicon encompassing these two 3’ CNEs and the intervening sequence (1.34 Kb) was PCR-amplified from genomic DNA. Forward primer: AAGATTGGAATGGAATGTCT. Reverse primer: GCATTTTCGCCTTTGCATCA.

The *Tg(evx1:EGFP)*^*SU1*^ line was generated by cloning this 3’ CNE amplicon into the pDONR^TM^P4-P1R vector from Invitrogen using Gateway technology [42, 43]. The final construct was assembled using the pENTR*basegfp* plasmid and the pCSDest2 vector [44]. This resulted in a vector containing *Tol2:1.3Kb 3’ zfish evx1:ßcarp minimal promoter:EGFP:Tol2*.

The same 3’ CNE amplicon was used to generate the *Tg(evx1:EGFP)*^*SU2*^ line. In this case, the *GAL4VP16;UAS-EGFP* cassette was taken from the *pBGAL4VP16;UAS-EGFP* plasmid [45] and cloned into a middle entry vector from Invitrogen [42, 43]. An oligonucleotide containing the *cfos* minimal promoter sequence [46] plus 17 bp of the 5’ arm of *GAL4* was synthesized and used with a RVeGFPAttb2 primer to PCR amplify the *GAL4VP16;UAS*-*EGFP* cassette.

FWcfosGAL4VP16 primer: CACTCATTCATAAAACGCTTGTTATAAAAGCAGTGGCTGCGGCGCCTCGTACTCCAACCGCATCTGCAGCGAGCAACTGAGAAGCCAAGACTGAGCCGGCGGCCTTTGTACAAAAAAGCAG

RVeGFPAttb2 primer: GGGGACCACTTTGTACAAGAAAGCTGGGTTTACTTGTACAGCTCGTCCA.

This PCR product was used to generate a second PCR product using the primer FWattB1cfos: GGGGACAAGTTTGTACAAAAAAGCAGGCTCACTCATTCATAAAATCGCTT and the RVeGFPAttb2 primer.

The final amplicon was cloned into pDONR™221 using gateway technology. This middle entry vector was used to generate a final vector containing *Tol2:1.3Kb 3’ zfish evx1:cfos minimal promoter:GAL4VP16;UAS-EGFP:Tol2.*

Each of these plasmids was separately co-injected with transposase mRNA into 1-2 cell embryos as described by [47]. Embryos were raised to adulthood and out-crossed to identify founders. In each case one stable transgenic line was generated. The *Tg(evx1:EGFP)*^*SU2*^ or *Tg(1.3 kb evx1:cfos:GAL4-UAS:EGFP)* line has the advantage that it contains a GAL4-UAS cassette to amplify EGFP expression. This facilitates visualization of axons. However, this line has a slightly more variegated expression than the *Tg(evx1:EGFP)*^*SU1*^ or *Tg(1.3Kb evx1:ßcarp:EGFP)* line, presumably because of stochastic silencing of the construct due to the GAL4-UAS sequences [48]. In contrast the *Tg(evx1:EGFP)*^*SU1*^ line labels all V0v cells more consistently, but the EGFP expression is slightly weaker and we were never able to obtain *evx1*^*-/-*^*;evx2*^*-/-*^ double mutant embryos that contained this transgene, even though we could obtain *evx1*^*-/-*^*;evx2*^*+/+*^ and *evx1*^*-/-*^*;evx2*^*+/-*^ embryos. This suggests that the *Tg(1.3Kb evx1:ßcarp:EGFP)* construct integrated close to the WT *evx2* allele.

### Morpholino injection

Approximately 5 nl of a 1:1 combination of two Evx2 ATG Morpholino antisense oligonucleotides (MOs) at 1.25 mg/ml each were injected into 1-2 cell embryos (evx2-1 MO: TTCTTTTCTTATCCTCTCCATCATG; evx2-2 MO: AATCCAAAGTCCCAGGGCTGGTGCT). In all cases, we confirmed that the MOs had completely knocked down Evx2 using immunohistochemistry for zebrafish Evx2.

### Expression profiling V0v cells

To determine which neurotransmitters V0v cells express and to identify additional transcription factors expressed by these cells, different combinations of spinal cord and trunk cells were extracted from live transgenic zebrafish embryos at 27 h using fluorescence activated cell-sorting (FACS). Prior to FACS, embryos were prim-staged, deyolked, dissected and dissociated as in [49, 50]. In all cases, the heads were removed to ensure that only trunk or spinal cord cells were collected. Pure populations of cells were obtained using combinations of the following transgenic lines: *Tg(elav13:EGFP), Tg(evx1:EGFP)*^*SU1*^, *Tg(pax2a:GFP), Tg(Xla.Tubb:DsRed* (formerly *Tg(NBT:DsRed))*, *Tg(vsx2:DsRed)* and *Tg(gata1:GFP)* [8, 51–54]. Trunk samples correspond to FAC-sorted trunk cells (spinal cord and other tissues). All neuron samples are EGFP-positive cells from *Tg(elav13:EGFP)* trunks. V0v neurons are EGFP-positive cells from *Tg(evx1:EGFP)*^*SU1*^ trunks. V1 neurons are double-positive EGFP-positive, DsRed-positive cells from *Tg(pax2a:GFP);Tg(Xla.Tubb:DsRed)* trunks. V2a neurons are double-positive DsRed-positive, EGFP-positive cells from *Tg(vsx2:DsRed);Tg(elavl3:EGFP)* trunks. V2b + KA neurons are double-positive EGFP-positive, DsRed-positive cells from *Tg(gata1:GFP);Tg(Xla.Tubb:DsRed)* trunks. Total RNA was extracted using an RNeasy Micro Kit (Qiagen, 74004). RNA quality and quantity was assayed on an Agilent 2100 Bioanalyser (RNA 6000 Pico Kit, Agilent, 5067-1513), before converting to fluorescently-labelled cDNA (Ovation Pico WTA System V2, Pico, 3302) and hybridizing to a custom-designed Agilent microarray (EMBL Genomics Core, Heidelberg). Details of this microarray will be described elsewhere, along with the characterization of additional genes identified from these analyses. Data pre-processing and normalization was performed using Bioconductor software (https://www.bioconductor.org/). Two-class eBayes and three-class ANOVA analyses were performed using GEPAS software (Tárraga, (2008)). All reported statistics were corrected for multiple testing (Benjamini and Hochberg (1995)).

### *in situ* hybridization

Embryos were fixed in 4 % paraformaldehyde and single and double *in situ* hybridizations were performed as previously described [55, 56]. RNA probes were prepared using the following templates, *dbx1a* and *dbx1b* [57], *evx1* [58], *evx2* [32], *eve1* [59], *pax2a, pax2b, pax8* [60] and *eng1b* [14]. To determine neurotransmitter phenotypes we used probes for genes that encode proteins that transport or synthesize specific neurotransmitters. A mixture of two probes (*glyt2a* and *glyt2b*) for *slc6a5* (previously called *glyt2*) was used to label glycinergic cells [61, 62]. *slc6a5* encodes for a glycine transporter necessary for glycine reuptake and transport across the plasma membrane. A mixture of two probes to *gad1b* (previously called *gad67,* probes used to be called *gad67a* and *gad67b*) and one probe to *gad2* (previously called *gad65*) was used to label GABAergic cells [61, 62]. *gad1b* and *gad2* encode for glutamic acid decarboxylases, necessary for the synthesis of GABA from glutamate. A mixture of *slc17a6b* (formerly called *vglut2.1)* and *slc17a6a* (formerly called *vglut 2.2)* probes was used to label glutamatergic cells [61, 62]. These genes encode proteins responsible for transporting glutamate to the synapse. In all of these cases, a mix of equal concentrations of the relevant probes was used [61, 62]. We also used *slc32a1* (formerly called *viaat)*, which encodes for a vesicular inhibitory amino acid transporter, to label all inhibitory cells [8].

The DNA template for the s*kor2* (ZDB-GENE-060825-57) probe was generated by PCR-amplifying the 3’ region of *skor2* from cDNA using a reverse primer containing a T3 promoter sequence at the 5’ end (indicated in italics below). Total RNA was extracted by homogenizing 50-100 mg of 27hpf wild-type zebrafish embryos in 1 mL of TRIzol reagent (Ambion, 15596-026). cDNA was synthesized using Bio-Rad iScript Reverse Transcription Supermix kit (Bio-Rad, 170-8891). A 50 μL PCR was assembled containing 5 μL cDNA and 1 unit of Phusion High-Fidelity DNA Polymerase (NEB, M0530L). PCR conditions were: 94 °C for 3 min followed by 35 cycles of 94 °C for 30 s, 56.5 °C for 30 s, 72 °C for 1.5 min and then a final extension step of 72 °C for 10 min. PCR product was purified by phenol:chloroform extraction. Forward primer: CGCAAGACGCTTTTTATCC

Reverse primer: *AATTAACCCTCACTAAAGGGA*AAATGGAGAGCTGCCTTTCAG.

ZFIN Identification numbers are provided for all genes in Additional file 1: Table S2.

### Immunohistochemistry

Primary antibodies used were rabbit anti-Evx2 (a kind gift from Dr Higashijima, described in Satou et al., 2012, raised against the first 168 amino acids of zebrafish Evx2, a region with no significant homology to zebrafish Evx1, 1:300), mouse anti-GFP (Roche Applied Science, 11814460001, 1:500), rabbit anti-GFP (Molecular Probes A6465, 1:500) and mouse anti-Pax2 (Covance PRB-276P 1:300). The Pax2 antibody recognizes both Pax2a and Pax2b in zebrafish [14]. Antibodies used for fluorescent *in situ* hybridization were mouse anti-Dig (Jackson ImmunoResearch 200-002-156, 1:5000) and rabbit anti-Flu (Invitrogen A889, 1:2500). These were detected with Invitrogen Tyramide kits #12 and #5. Secondary antibodies used were Alexa Fluor 568 goat anti-rabbit (Molecular Probes A11036, 1:500), Alexa Fluor 488 goat anti-rabbit (Molecular Probes A11034, 1:500) and Alexa Fluor 488 goat anti-mouse (Molecular Probes A11029, 1:500).

Embryos for immunohistochemistry were treated with acetone for 15 min (24 h embryos) or 20 min (30 h embryos) to permeabilize them, washed for 5 min in distilled water, then washed 2 x 10 min in PBS. Embryos were treated with Image-iT Signal Enhancer (Invitrogen, I36933) for 30 min, then incubated in block solution (2 % goat serum, 1 % BSA, 10 % DMSO and 0.5 % Triton) for 1 h at room temperature followed by incubation in primary antibody in fresh block solution at 4 °C overnight. Embryos were washed with PBT (PBS + 0.1 % Triton) for 2 h at room temperature and incubated with secondary antibody in block solution at 4 °C overnight. Embryos were then washed with PBT for at 2 h at room temperature and stored in 2 % DABCO (Acros Organics, AC11247-1000).

For 3,3’- diaminobenzidine (DAB) staining*,* after incubation with primary antibody, samples were incubated in fresh blocking solution with goat anti-rabbit IgG (Covance SMI-5030C, 1:200) at 4 °C overnight. Embryos were then washed with PBT for 2 h and incubated with rabbit PAP (Covance SMI-4010 L, 1:200) in block solution at 4 °C overnight. Embryos were then washed in PBT for 2 h. Staining was performed using Sigma Fast 3,3’- diaminobenzidine tablets (Sigma, D4293).

### Imaging

Embryos were mounted in 70 % glycerol, 30 % PBS and DIC pictures were taken using an AxioCam MRc5 camera mounted on a Zeiss Axio Imager M1 compound microscope. Fluorescent images were taken on a Zeiss LSM 710 confocal microscope. Images were processed using Adobe Photoshop software (Adobe, Inc) and Image J software (Abramoff et al., 2004). In some cases different focal planes were merged to show labeled cells at different medial lateral positions in the spinal cord.

### Cell counts and statistics

In all cases, cells counts are for both sides of a five-somite length of the spinal cord adjacent to somites 6-10. Most values are an average of at least 5 embryos. Exceptions are the *skor2* + *Tg(evx1:EGFP)* double-labeling experiments, the *skor2* + *Tg(slc17a6:EGFP)* double-labeling experiments and the *pax2a* and *eng1b in situ* hybridization results. In all of these cases 4 embryos were counted. Results were analyzed using the student’s *t*-test; Error bars indicate standard deviation.

## Results

### Zebrafish V0v cells express *evx1* and *evx2*

In mouse, *Evx1* is expressed in V0v cells and while double-labeling experiments have not yet been performed, the data suggest that *Evx2* is probably co-expressed by these same cells [23, 24, 27, 28]. Previous reports described *evx1* and *evx2* expression in a similar region of zebrafish spinal cord [32, 33] but didn’t determine whether these genes are co-expressed or the specific cell types that express them.

**Fig. 2:**
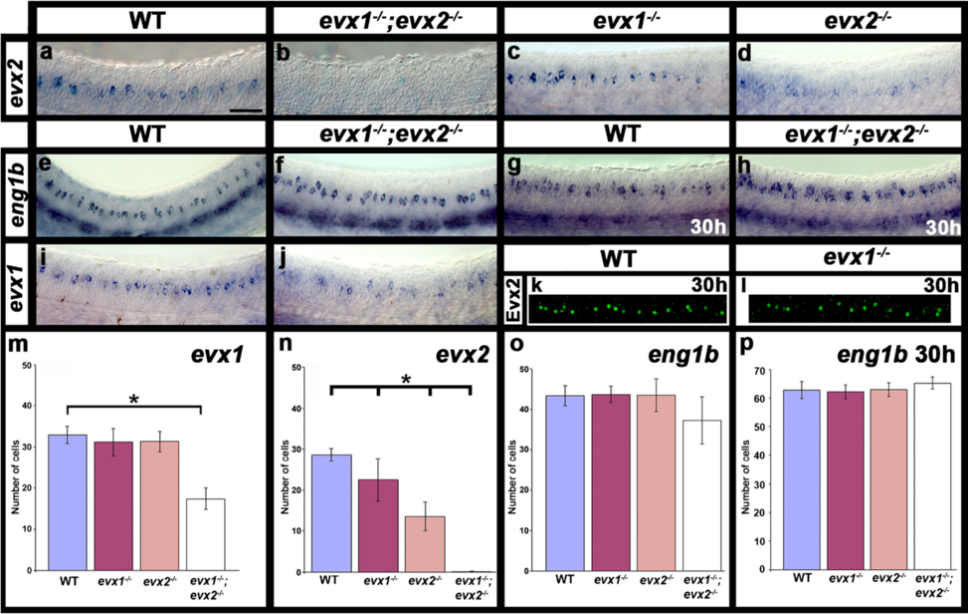
Expression of *evx* and *eng1b* genes in *evx1;evx2* double mutant embryos. Lateral views of zebrafish spinal cord at 24 h (**a**-**f**, **i** & **j**) or 30 h (**g**, **h**, **k **& **l **). Anterior left, dorsal up. **a**-**j** *in situ* hybridization for each gene indicated. **e**-**h** strong ventral expression is in muscle pioneer cells, expression in more individual dorsal cells corresponds to spinal cord V1 cells. **k** & **l** immunohistochemistry for Evx2. **m**-**p** Average number of cells (y-axis) expressing indicated marker in spinal cord region adjacent to somites 6-10 in WT embryos and *evx1* and *evx2* single and double mutants (x-axis) at 24 h (**m**-**o**) or 30 h (**p**). Values are shown as mean +/- standard deviation (values are provided in Table 1). There are no *evx2*-positive cells in the double mutants (**n**). In each case at least 5 embryos were counted, except for *eng1b* where 4 embryos were counted. Statistically significant differences (P < 0.05) from WT values are indicated with brackets and stars. P values for these and other comparisons (e.g differences between single and double mutants) are provided in Table 1. Scale bar: 50 μm (**a**-**l**)

To address these questions, we performed single and double *in situ* hybridization experiments and found that zebrafish *evx1* and *evx2* are co-expressed in an intermediate region of the dorso-ventral axis of the spinal cord (Figs. 1g and 2a & i). We further confirmed that *evx1* and *evx2* are co-expressed in zebrafish spinal cord using an EGFP line, *Tg(evx1:EGFP)*^*SU1*^. We constructed this line using enhancer sequences identified downstream of *evx1* (see methods & Fig. 1d). We confirmed that the stable line recapitulates endogenous *evx1* expression (Fig. 1f) and also shows co-expression of EGFP and Evx2 protein (Fig. 1e). Interestingly, at 27 h, all of the cells that express Evx2 also express EGFP (and hence *evx1*) (Fig. 1e), however, a few cells express EGFP but not Evx2. Similarly, at 24 h, a few cells express *evx1* but not *evx2* (Fig. 1g). This is consistent with earlier reports that suggest that *evx1* may be expressed in the spinal cord slightly earlier than *evx2* [32, 33], although we cannot rule out the possibility that there is a very small subset of *evx1-*expressing V0v cells that do not express *evx2*.

V0 cells develop from the p0 progenitor domain, which expresses *dbx1* [28, 63]. Therefore, to confirm that *evx1/2*-expressing cells are V0v cells we performed EGFP immunohistochemistry and *in situ* hybridization for *dbx1a* and *dbx1b* in *Tg(evx1:EGFP)*^*SU1*^ embryos. We found that zebrafish *evx* genes are expressed lateral to cells expressing both of these *dbx1* genes, as would be predicted for cells developing from the p0 domain. In addition, *dbx1a* and *dbx1b* expression persists in some EGFP-positive cells (Fig. 1h), suggesting that these genes continue to be expressed by V0v cells for a short while after they become post-mitotic.

**Fig. 3:**
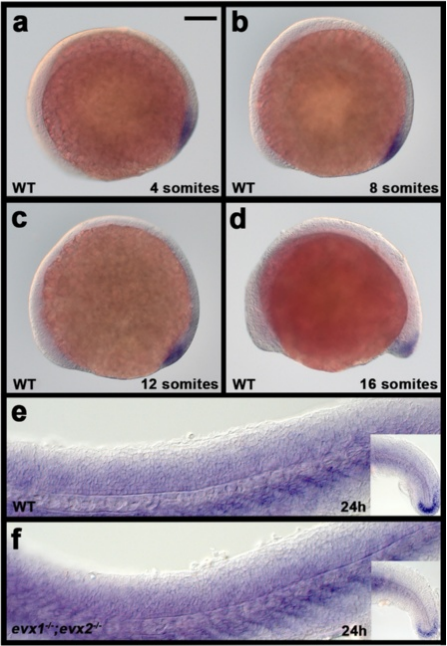
*eve1* is not expressed in zebrafish spinal cord in WT or *evx1;evx2* double mutant embryos. Lateral views of *in situ* hybridization for *eve1* in 4-somite (**a**), 8-somite (**b**), 12-somite (**c**), 16-somite (**d**) and 24 h (**e & f**) embryos. **a**-**e** WT; (**f**) *evx1;evx2* double mutant. Anterior is left and dorsal up. Expression is seen in the tail bud region but not the spinal cord. Embryos in (**e & f**) were over-stained to check that there was no weak expression in the spinal cord. The only specific staining seen was at the end of the tail (inset in right bottom corner). The rest is background staining from over-staining. Scale bar: 100 μm

Zebrafish also have a third *evx* gene, called *eve1*, but earlier studies suggested that this gene is not expressed in the spinal cord [26, 32, 59, 64]. We confirmed this by examining *eve1* expression at multiple stages of spinal cord development (every two somites from 2-somites - 24 h; Fig. 3 and data not shown). In all cases we never saw any spinal cord expression, only expression in the developing tailbud. To check whether *eve1* expression is altered in the absence of Evx1 and/or Evx2 we also examined expression in *evx1;evx2* double mutants. However, we saw no change in *eve1* expression in these double mutants (Fig. 3f). Therefore, this gene is not considered further in this paper. We also confirmed that no additional *evx* genes exist in zebrafish (Additional file 1: Results and Figure S1).

### Zebrafish V0v cells develop into commissural ascending interneurons

**Fig. 4:**
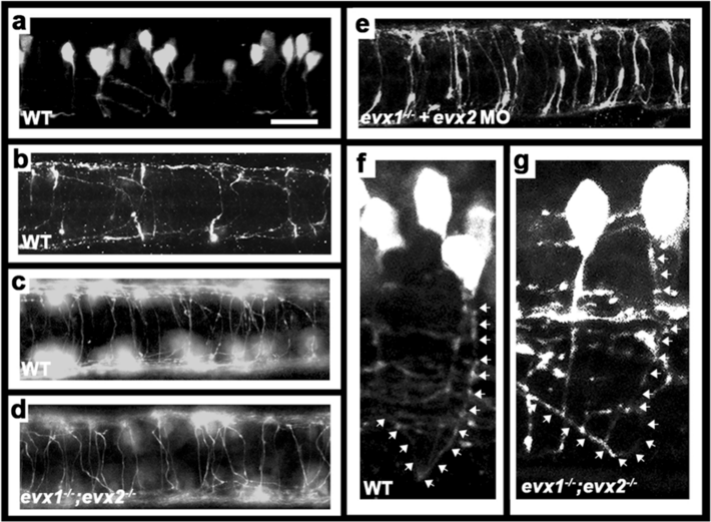
V0v cells develop into CoSA interneurons. Immunohistochemistry for EGFP in *Tg(evx1:EGFP)* ^*SU1*^ (**a**, **b** & **e**) or *Tg(evx1:EGFP)* ^*SU2*^ (**c**, **d**, **f** & **g**) embryos.^*.*^ **a**, **f** & **g** lateral views with dorsal up and anterior left of spinal cord at 27 h (**a**) or 48 h (**f** & **g**). **b**-**e** dorsal views with anterior left of zebrafish spinal cord at 30 h (**b**) or 48 h (**c**-**e**). **a**-**c** & **f** WT, (**d** & **g**) *evx1;evx2* double mutant, (**e**) *evx1* mutant injected with *evx2* morpholino. **b** & **c** show increasing number of commissural axons crossing the spinal cord as development proceeds. **d** & **e** demonstrate that V0v axons are still clearly commissural in the absence of Evx1 and Evx2. **f** & **g** show magnified views of commissural ascending V0v axons. White arrows (drawn slightly to the right of the axon so that EGFP expression is still visible) indicate ascending axon trajectories. Scale bar: 50 μm (**a**-**e**) and 15 μm (**f** & **g**)

In mouse, V0v cells develop commissural axons that ascend (grow rostrally) for one to four somite segments [23]. We used both the *Tg(evx1:EGFP)*^*SU1*^ transgenic line and an additional transgenic line that has stronger expression in V0v cell axons, *Tg(evx1:EGFP)*^*SU2*^ (see methods), to examine the morphology of zebrafish V0v cells. We found that by 27-30 h, almost all of the cells have extended their axons ventrally and have at least started to cross the midline to the other side of the spinal cord (Fig 4a & b). By 48 h, most of the cells have reached the other side of the spinal cord and have turned towards the head, giving them a clear commissural ascending, or CoSA [65, 66], morphology (Fig. 4c & f).

### V0v cells are glutamatergic

**Fig. 5:**
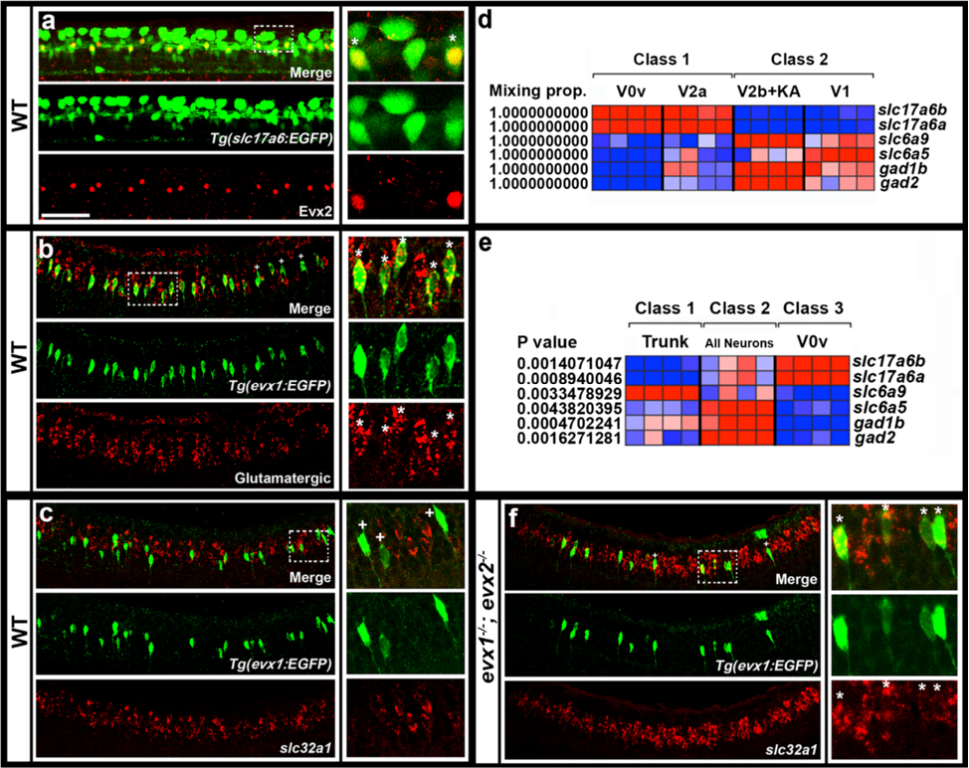
Neurotransmitter phenotypes of V0v cells. Lateral views of spinal cord at 27 h (**a** & **b**) or 30 h (**c** & **f**). All panels contain merged and single channel views. Smaller images on RHS are single confocal planes of white box regions. Double and single-labeled EGFP-positive cells are indicated in single confocal planes with stars and crosses respectively. **a** EGFP (green) and Evx2 (red) expression in *Tg(slc17a6:EGFP)* embryo. All Evx2-positive cells co-express EGFP. **b** EGFP (green) and glutamatergic marker (*slc17a6b & slc17a6a*; red) expression in *Tg(evx1:EGFP)* ^*SU1*^ embryo. Occasional single-positive cells are indicated with crosses. These may be expressing glutamatergic markers at levels too low to detect (*slc17a6* probes are weak in double-labeling experiments). Remaining cells are double-labeled. **c** & **f** EGFP (green) and *slc32a1* (red) expression in *Tg(evx1:EGFP)* ^*SU2*^ WT (**c**) and *evx1;evx2* double mutant (**f**) embryos. No V0v cells are inhibitory in WT embryos, but most V0v cells are inhibitory in double mutants (occasional single-labeled cells are indicated with a cross in F). (**d** & **e**) Relative expression profiles of genes (names on right) indicative of neurotransmitter fates at 27 h. Columns represent individual microarray experiments. Rows indicate relative expression levels as normalized data transformed to mean of zero and standard deviation of +1 (highly-expressed, red) to -1 (weakly/not expressed, blue) sigma units. For details of how cells were isolated see methods. **d** Two-class eBayes comparison of excitatory (class 1) versus inhibitory (class 2) cells. Mixing proportion measures posterior probability, or likelihood that genes are differentially expressed (1 = highest probability of differential expression). **e** Three-class ANOVA comparison of V0v cells (class 3) versus trunk cells (class 1) and all post-mitotic neurons (class 2). P values test hypothesis that there is no differential expression between the 3 classes. V0v cells express glutamatergic markers *slc17a6a* and *slc17a6b* and do not express glycinergic or GABAergic markers in both comparisons. Scale bar: 50 μm (**a**-**c** & **f**)

In wild-type mouse spinal cords, V0 cells develop into both inhibitory (glycinergic or GABAergic) and excitatory (glutamatergic) interneurons [27, 28, 36], but when we started this project the neurotransmitter phenotype of V0v cells had not been established. Zebrafish have both excitatory and inhibitory CoSA interneurons [61, 62], so we could not infer the neurotransmitter properties of V0v cells from their morphology alone. Therefore, we performed double-labeling experiments to establish the neurotransmitter fates of these cells. Double immunohistochemistry for Evx2 and EGFP in the *Tg(slc17a6:EGFP)* line which labels glutamatergic interneurons in the zebrafish spinal cord [37, 67] revealed that all of the Evx2-positive cells are glutamatergic (Fig. 5a). As expected, not all of the glutamatergic cells express Evx2, since there are several excitatory cell types in the zebrafish spinal cord and only V0v cells express Evx2. In addition, *in situ* hybridization for *slc17a6 (vglut)* genes*,* markers of glutamatergic cells (see methods for more details) combined with EGFP immunohistochemistry in *Tg(evx1:EGFP)*^*SU1*^ embryos also confirmed that *evx1*-expressing cells are glutamatergic (Fig. 5b). We also examined if *evx1* is co-expressed with markers of any other spinal cord neurotransmitter fates. Using double *in situ* hybridization we found no co-expression between *evx1* and markers for glycinergic or GABAergic markers (data not shown). We also observed no double-labeled cells when we performed EGFP immunohistochemistry and *in situ* hybridization for s*lc32a1* (formerly called *viaat*) which labels all inhibitory neurons [8, 68, 69] in *Tg(evx1:EGFP)*^*SU2*^ embryos (Fig. 5c).

Consistent with these analyses, when we FAC-sorted and expression profiled EGFP-labeled V0v cells using the *Tg(evx1:EGFP)*^*SU1*^ line we found that these cells express markers of glutamatergic fates (*slc17a6a* (formerly called *vglut2.2*) and *slc17a6b* (formerly called *vglut2.1*)) and do not express either glycinergic markers (*slc6a9* (formerly called *glyt1*) or *slc6a5* (formerly called *glyt2*)) or GABAergic markers (*gad1b* or *gad2*) (Fig. 5d & e).

### Zebrafish V0v cells do not express Pax2

**Fig. 6:**
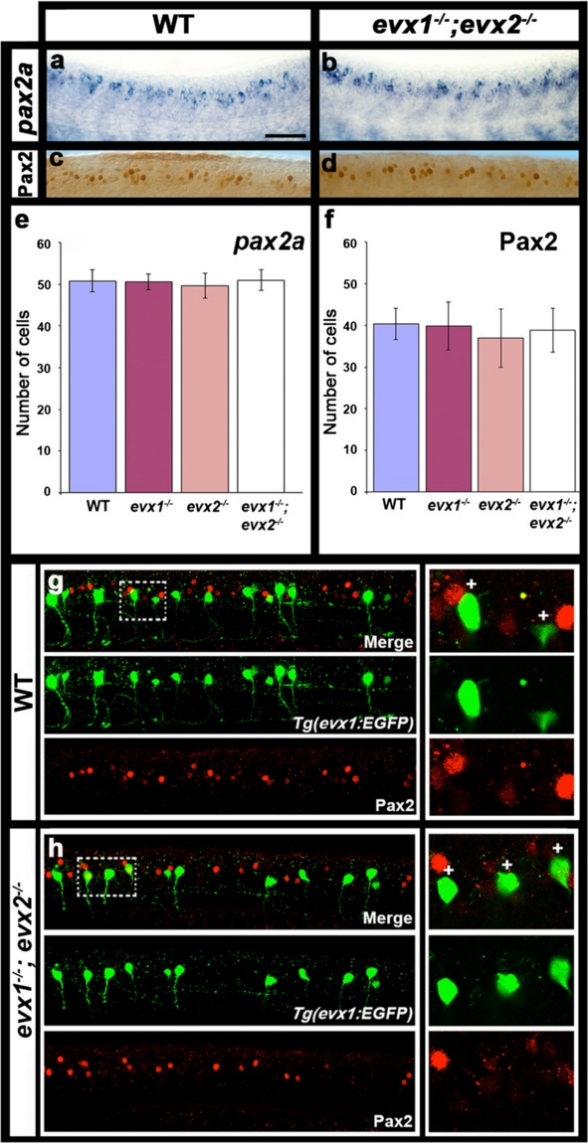
V0v cells do not express Pax2 in WT or *evx1;evx2* double mutant embryos. Lateral views of zebrafish spinal cord at 24 h (**a**-**d**) or 30 h (**g** & **h**). Anterior left, dorsal up. **a** & **b** *in situ* hybridization for *pax2a*. **c** & **d** immunohistochemistry for Pax2. The Pax2 antibody recognizes both Pax2a and Pax2b. **e** & **f** Average number of cells (y-axis) expressing these markers (indicated in each case) in spinal cord region adjacent to somites 6-10 in WT embryos and *evx1* and *evx2* single and double mutants (x-axis). Values are shown as mean +/- standard deviation (values are provided in Table 1). In each case at least 5 embryos were counted, except for *pax2a* where 4 embryos were counted. P values for all comparisons are provided in Table 1. **g** & **h** EGFP (green) and Pax2 (red) expression in *Tg(evx1:EGFP)* ^*SU2*^ WT (**g**) and *evx1;evx2* double mutant (**h**) embryos. No V0v cells express Pax2 in either case. Panels on RHS are magnified single-confocal-plane views of white dotted rectangle regions in panels G and H respectively. White crosses indicate single-positive GFP cells. Scale bar: 50 μm (**a**-**d**) & 40 μm (**g**-**h**)

As discussed above, Pax2 is an important regulator of inhibitory spinal cord fates [14–21]. Given that we had shown that V0v cells are excitatory (glutamatergic) and we had not observed any inhibitory V0v cells (Figs 5a-e), we would not predict that V0v cells would express Pax2. However, the literature contains contradictory evidence as to whether *evx1* and *evx2* are co-expressed with *pax2* in the spinal cord [35, 58, 70, 71]. To resolve this issue, we performed double-labeling experiments for *evx1* and *pax2* using several complementary approaches. These included *in situ* hybridization for *evx1* and immunohistochemistry for Pax2, EGFP immunohistochemistry and *in situ* hybridization for *pax2a*, *pax2b* and *pax8* (three highly-related *pax* genes that are co-expressed in zebrafish spinal cord cells [14]) in *Tg(evx1:EGFP)*^*SU2*^ embryos and double immunohistochemistry for EGFP and Pax2 in *Tg(evx1:EGFP)*^*SU2*^ embryos. In all cases, we observed no double-labeled cells, suggesting that *evx1* and *evx2* are not co-expressed with *pax2/ pax8* genes in zebrafish spinal cord (Fig. 6g and data not shown). These analyses complement those of Satou and colleagues [36] who recently reported that inhibitory V0 cells, which presumably correspond to V0_D_ cells, express Pax2, but Evx2-expressing cells do not. Taken together, these data strongly suggest that V0v cells do not express *pax2* genes.

### *evx2*^*sa140*^ is a null allele

To identify the functions of *evx1* and *evx2* in zebrafish V0v cells we used *evx1* and *evx2* mutants (see Methods). Our previous analyses suggest that *evx1*^*i232*^ is a null allele [39]. The *evx2*^*sa140*^ mutation introduces a premature stop codon just before the homeodomain, suggesting that if a truncated protein is synthesized it will have no DNA binding activity. However, it is possible that a truncated protein might retain some function in the embryo. To determine if any Evx2 protein is made in mutant embryos, we used an Evx2 antibody that was made against the first 168 amino acids of zebrafish Evx2 (which corresponds exactly to the region upstream of the premature stop codon in the *evx2*^*sa140*^ mutant allele). We found that all of the WT (21/51) and heterozygous embryos (20/51) had Evx2 antibody staining but all of the homozygous mutants (10/51) did not (Fig. 1c). This strongly suggests that the *evx2* mutant allele does not produce any protein and is a null allele.

### *evx2*^*sa140*^ homozygous mutants are not viable

Unlike the zebrafish *evx1* mutant, which is homozygous viable [39], we never identified an adult *evx2* homozygous mutant (n = 262 fish from incrosses of heterozygous *evx2* fish, P < 0.0001 using chi-squared test). However, we did obtain fish homozygous for *evx1* and heterozygous for *evx2* (17 fish identified from a total of 191 fish from incrosses of heterozygous double mutants; P = 0.13 using chi-squared test). Our analyses of incrosses from identified *evx2* heterozygous fish suggest that *evx2* mutant embryos have no obvious morphological defects for the first few days of development but that most of them die by larval stages (see Additional file 1: Results).

### V0v cells still form in *evx1;evx2* double mutants

In mouse *Evx1* mutants, expression of Evx2 is lost and there is an increase in the number of cells expressing the V1 marker *En1*. In addition, many of the cells that would normally have expressed *Evx1* develop axon trajectories similar to V1 cells. Most strikingly their axons change from being commissural to ipsilateral [23]. This suggests that in the absence of Evx1, most mouse V0v cells transfate to V1 cells.

In contrast, we found that in zebrafish *evx1* mutants there is only a small reduction in the number of cells expressing *evx2* RNA and Evx2 protein in the spinal cord (approximately a 22 % reduction for *evx2* at 24 h and a 32 % reduction for Evx2 at 30 h; Fig. 2c, l & n; Table 1), although expression of *evx2* RNA is lost completely in *evx1;evx2* double mutants (Fig. 2b & n; Table 1). In contrast, there is no difference in *evx1* expression in *evx1* or *evx2* single mutants when compared to WT siblings, although approximately 47 % of V0v cells lose expression of *evx1* in double mutants (Fig. 2j & m; Table 1). Consistent with the down-regulation of *evx1* in double mutants, we observe a 30 % reduction in the number of EGFP-labeled V0v cells in double mutant *Tg(evx1:EGFP)*^*SU2*^ embryos (22.9 +/- 3.4 cells in WTs; 16.0 +/- 5.5 cells in double mutants). However, strikingly, most V0v cells are still labeled with EGFP and these cells have a normal commissural ascending CoSA morphology (Fig. 4d & g). Unfortunately, we were never able to identify double mutant embryos that carried the *Tg(evx1:EGFP)*^*SU1*^ transgene, suggesting that this transgene probably integrated in the vicinity of the WT *evx2* allele (see methods). However, consistent with our results using the *Tg(evx1:EGFP)*^*SU2*^ transgenic line, when we injected *evx2* ATG morpholinos, at a concentration that eliminates Evx2 protein, into *evx1* single mutant *Tg(evx1:EGFP)*^*SU1*^ embryos, we also observed EGFP-labeled V0v cells with normal CoSA axon trajectories (Fig. 4e).

**Table 1:** Number of cells expressing particular genes and proteins in WT and mutant embryos

Marker	Stage	WT	*evx1* mutants	P^a^	*evx2* mutants	P^b^	Double mutants	P^c^	P^d^	P^e^
*evx1*	24 h	33.0 + /-2.0	31.0 + /-3.3	0.32	31.3 + /-2.5	0.56	17.4 + /-2.6	**<0.01**	**<0.01**	**<0.01**
*evx2*	24 h	28.6 + /-1.6	22.4 + /-50	**0.02**	13.5 + /-3.5	**<0.01**	0.00	**<0.01**	**<0.01**	**<0.01**
Evx2	30 h	33.6 + /-5.2	23.0 + /-4.6	**0.01**	N.D		N.D			
*eng1b*	24 h	43.3 + /-2.5	43.6 + /-2.0	0.80	43.5 + /-4.0	0.94	37.2 + /-6.0	0.08	0.07	0.10
*eng1b*	30 h	63.0 + /-3.0	62.0 + /-2.5	0.63	63.0 + /-2.5	0.92	65.2 + /-2.0	0.15	0.06	0.15
*slc17a6 (vlgut)*	24 h	89.0 + /-10.0	71.3 + /-4.3	**<0.01**	74.6 + /-8.0	**0.01**	60.8 + /-10	**<0.01**	0.08	**0.04**
s*lc32a1 (viaat)*	24 h	157.4 + /-8.0	157.3 + /-10.0	0.75	158.2 + /-4.0	0.86	178.6 + /-7.0	**<0.01**	**<0.01**	**<0.01**
*gads* (GABAergic)	24 h	50.0 + /-3.0	49.0 + /-4.0	0.59	51.6 + /-2.6	0.31	50.6 + /-2.2	0.66	0.38	0.5
*slc6a5 (glyt2a/glyt2b)*	24 h	81.0 + /-3.3	93.0 + /-9.0	**<0.01**	87.6 + /-4.5	**0.02**	109 + /-10.0	**<0.01**	**0.02**	**<0.01**
*pax2a*	24 h	50.6 + /-2.6	50.4 + /-2.0	0.86	49.5 + /-30	0.55	50.8 + /-2.5	0.91	0.75	0.49
Pax2	24 h	40.3 + /-3.8	39.8 + /-5.7	0.83	36.9 + /-6.9	0.23	38.8 + /-5.3	0.41	0.68	0.52
*skor2*	30 h	24.3 + /-3.0	15.0 + /-5.0	**<0.01**	19.8 + /-4.0	0.07	0.0	**<0.01**	**<0.01**	**<0.01**
*skor2* Total cell counts	30 h	46.7 + /-3.7	38.7 + /-3.3	**<0.01**	42.4 + /-1.7	**0.04**	22.6 + /-1.7	**<0.01**	**<0.01**	**<0.01**

Numbers of cells expressing particular markers (first column on left) in spinal cord region adjacent to somites 6-10 and P values of comparisons between embryos with different genotypes. Values are shown as the mean from at least 5 different embryos +/- standard deviation, except for *pax2a* and *eng1b* where 4 embryos were counted. P values are from student’s *t*-tests. Statistically significant (P < 0.05) values are indicated in bold. P^a^ compares *evx1* single mutants with WT embryos, P^b^ compares *evx2* single mutants with WT embryos, P^c^, P^d^ and P^e^ are for comparisons between double mutant embryos and WT (P^c^), *evx1* single mutant (P^d^) and *evx2* single mutant (P^e^) embryos respectively. Mean cell count values are provided to one decimal place and *P* values to two decimal places. For *skor2,* two sets of values are provided: just the ventral domain of expression and both the ventral and dorsal domains of expression (total cell counts)

Consistent with this persistence of V0v cells, we also found no change in the number of *eng1b*-expressing spinal cord cells in *evx1* and *evx2* single or double mutants compared to WT embryos at 24 h. If anything, we observed a slight reduction in the number of *eng1b* cells in the double mutants, although this was not statistically significant (Fig. 2f & o, Table 1). To further confirm that V0v cells were not adopting a V1 fate and turning on *eng1b* expression, we repeated this experiment at 30 h. We still found no change in *eng1b* expression in either single or double mutants when compared to WT embryos (Fig. 2h & p, Table 1).

Taken together, these results suggest that V0v cells are not transfating into V1 cells in zebrafish, even in the absence of both Evx1 and Evx2. Instead at least most of these cells are maintaining their V0v identities. In addition, these data suggest that Evx1 and Evx2 act partially redundantly to maintain each other’s expression, although only *evx2* expression requires Evx1/Evx2 activity as more than half of V0v cells still express *evx1* in *evx1;evx2* double mutants.

### Evx1 and Evx2 are required for *skor2* expression in V0v cells

**Fig. 7:**
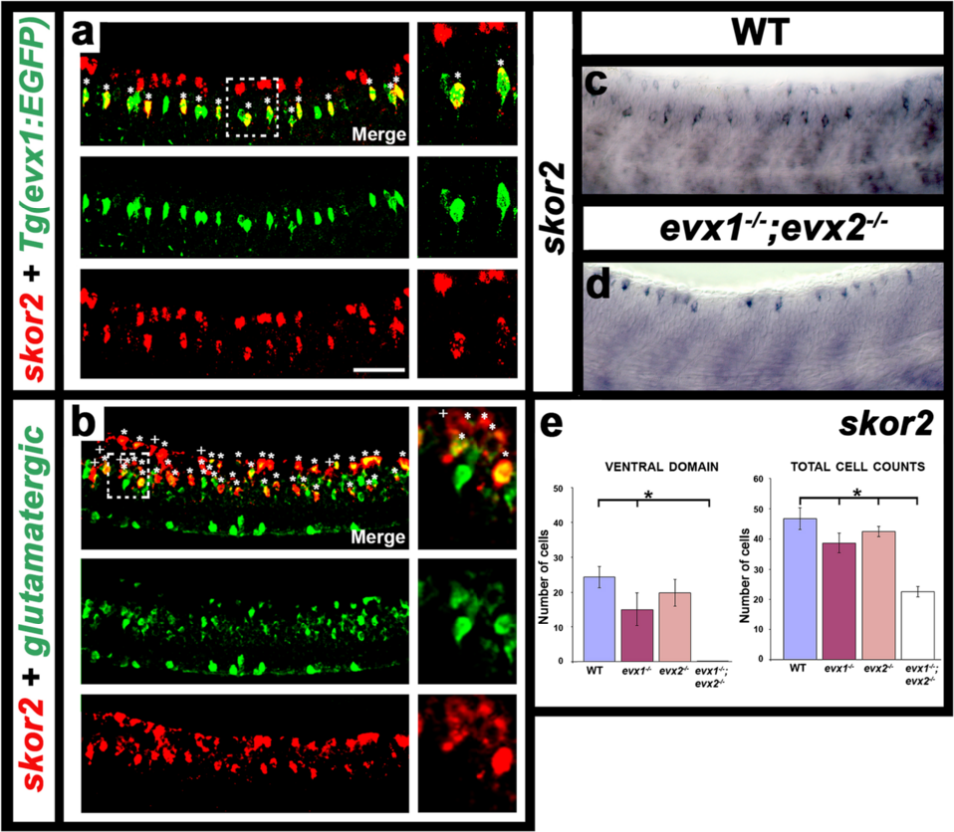
*skor2* is expressed by V0v cells and this expression is lost in *evx1;evx2* double mutants. Lateral views of zebrafish spinal cord at 27 h (**a**) and 30 h (**b**-**d**). Anterior left, dorsal top. **a** & **b** Merged images on top followed by single-channel views. Panels on RHS are single confocal planes of white dashed-box regions. Stars indicate double-positive cells. **a** Expression of *skor2* (red) and EGFP (green) in *Tg(evx1:EGFP)* ^*SU1*^ embryo. In this example, all ventral *skor2*-expressing cells co-express EGFP. On average, 97 % of ventral *skor2*-expressing cells co-express EGFP (73/75 cells counted in 4 embryos). In contrast, about 57.5 % of V0v cells co-express *skor2* (73/127 cells counted in 4 embryos). **b** Expression of *skor2* (red) and EGFP (green) in *Tg(slc17a6:EGFP)* embryo that labels glutamatergic cells. Crosses indicate cells that are only clearly positive for *skor2*. On average 93.5 % of *skor2*-expressing cells co-express EGFP (201/215 cells counted in 4 embryos). As there is usually a delay in EGFP expression it is possible that all ventral *skor2*-expressing cells are excitatory V0v cells, as the small number of ventral *skor2*-positive EGFP-negative cells may be just starting to express EGFP. **c** & **d** Expression of *skor2* (blue) in both WT (**c**) and *evx1;evx2* double mutant (**d**) embryos. The ventral row of *skor2* expression is lost in double mutants. **e** Average number of cells (y-axis) expressing *skor2* in spinal cord region adjacent to somites 6-10 in WT embryos and *evx1* and *evx2* single and double mutants (x-axis) at 30 h. Results are shown for the ventral (V0v) domain of *skor2* expression and for the whole *skor2* expression domain (total cell counts). Values are mean +/- standard deviation (also see Table 1). In each case at least 5 embryos were counted. Statistically significant differences (P < 0.05) from WT values are indicated with stars. P values for all comparisons are provided in Table 1. Scale bar: 50 μm (**a** & **b**); 40 μm (**c** & **d**)

To identify additional transcription factors that might be required for specification of V0v functional characteristics, we expression-profiled FAC-sorted V0v cells (see Methods; [50]). From these analyses, we identified *skor2* as a transcription factor gene potentially expressed by V0v neurons. Our subsequent *in situ* hybridization experiments demonstrated that *skor2* has two clear domains of spinal cord expression, a ventral domain and a more dorsal domain (Fig. 7c). Double labeling experiments show that in the ventral domain, at least most of the *skor2-*expressing cells are V0v cells (Fig. 7a). On average, 97 % of ventral *skor2*-expressing cells co-express EGFP in *Tg(evx1:EGFP)*^*SU1*^ embryos (73/75 cells counted in 4 embryos). Given this high number of double positive cells and the fact that there is usually a delay in EGFP expression it is possible that all of the ventral *skor2*-expressing cells are V0v cells. Interestingly, double labeling experiments with *skor2* and *Tg(slc17a6:EGFP)* demonstrated that both the ventral and dorsal *skor2*-expressing cells are excitatory cells (Fig. 7b), suggesting that Skor2 may play in role in specifying excitatory fates. As *skor2* is expressed by V0v cells, we tested whether it is regulated by Evx1 and Evx2. We found that the number of cells expressing *skor2* in the ventral spinal cord is reduced in *evx1* and *evx2* single mutants compared to WT embryos. More strikingly, ventral *skor2* expression is completely abolished in double *evx1;evx2* mutants (Figs 7d & e; Table 1), demonstrating that Evx1 and Evx2 are required, partially redundantly for *skor2* expression in V0v cells. In contrast, there was no change in the dorsal expression of *skor2* (Fig. 7d & e; Table 1).

### Evx1 and Evx2 are required to specify the glutamatergic fates of V0v cells

**Fig. 8:**
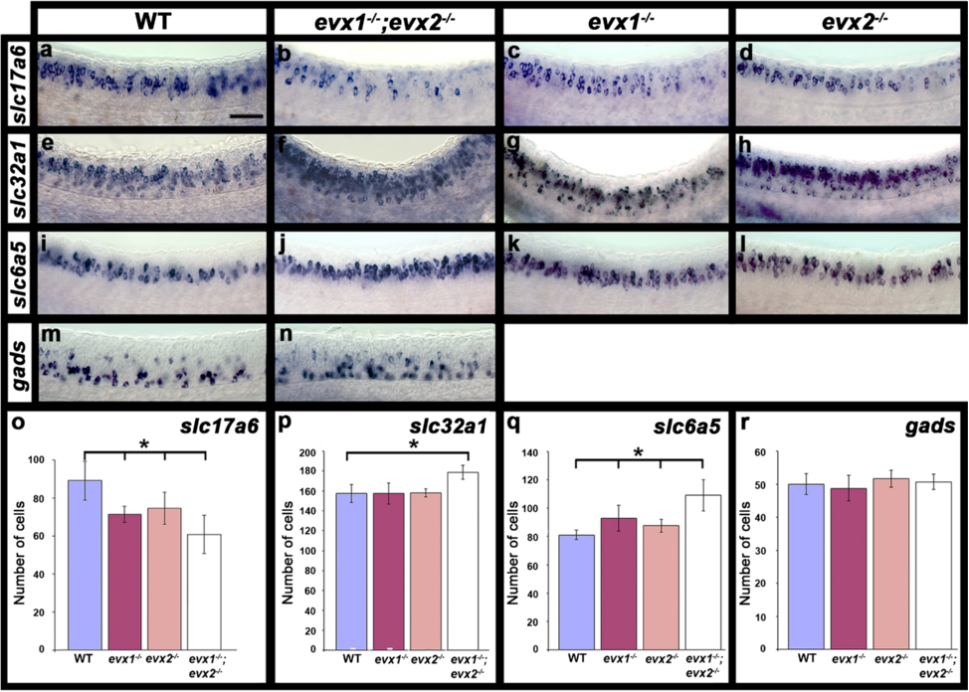
Neurotransmitter phenotypes in *evx1;evx2* double mutant embryos. Lateral views of zebrafish spinal cord at 24 h (**a**-**n**). Anterior is left, dorsal up. *in situ* hybridization for gene or genes indicated. *slc17a6* (**a**-**d**) corresponds to a mix of *slc17a6b* and *slc17a6a* probes that label glutamatergic cells; *slc32a1* (**e**-**h**, formerly called *viaat*) labels all inhibitory cells; *slc6a5* (**i**-**l**) labels glycinergic cells and *gads* (**m & n**) corresponds to a mix of *gad1a* and *gad2* probes that labels GABAergic cells (see methods for more details). **o**-**r** Average number of cells (y-axis) expressing these markers (indicated in each case) in spinal cord region adjacent to somites 6-10 in WT embryos and *evx1* and *evx2* single and double mutants (x-axis) at 24 h. Values are shown as mean +/- standard deviation (values are provided in Table 1). In each case at least 5 embryos were counted. Statistically significant differences (P < 0.05) from WT values are indicated with brackets and stars. P values for all comparisons are provided in Table 1. Scale bar: 50 μm (**a**-**n**)

Given that V0v cells still form in the absence of Evx1 and Evx2 function and their axon trajectories appear to be unaffected, but expression of a novel excitatory cell marker *skor2* is lost, we decided to test if V0v cell neurotransmitter phenotypes were changed. When we examined the expression of *slc17a6 (vglut*) genes in embryos from a cross of *evx1;evx2* heterozygous parents, we saw a significant reduction of glutamatergic cells in both of the single mutants when compared to WT embryos and an even more severe reduction in double mutants (Fig. 8a-d & o; Table 1). These data indicate that Evx1 and Evx2 act partially redundantly to specify the glutamatergic phenotype of V0v cells. Strikingly, the number of glutamatergic cells lost in the double mutant (approximately 28 cells in the spinal cord region adjacent to somites 6-10) is equivalent to the number of V0v cells in that region of the spinal cord (approximately 29 cells express *evx2* and 33 cells express *evx1* in this region of the WT spinal cord at this stage; see Table 1), suggesting that probably all of the V0v cells have lost their glutamatergic phenotype.

### V0v cells become inhibitory in *evx1;evx* double mutant embryos

Given that V0v cells lose their excitatory phenotype in *evx1;evx2* double mutants, we asked whether they are acquiring an inhibitory neurotransmitter fate instead. When we examined expression of s*lc32a1,* which is expressed by all inhibitory neurons [8, 68, 69], there was no significant difference in the number of cells expressing this gene between either of the single mutants and WT embryos (Fig. 8e, g, h & p, Table 1). However, interestingly, there was a significant increase (approximately 21 cells) in the number of *slc32a1-*expressing cells in *evx1;evx2* double mutants (Fig. 8f & p, Table 1). This suggests that Evx1 and Evx2 act redundantly to repress the inhibitory fate in V0v cells.

To further confirm that V0v cells are switching to an inhibitory fate, we performed *in situ* hybridization for *slc32a1* plus immunohistochemistry for EGFP in embryos from a cross of double heterozygous parents that carry the *Tg(evx1:EGFP)*^*SU2*^ transgene. In WT embryos we see no co-expression of *slc32a1* and EGFP (Fig. 5c). However, in double mutant embryos most V0v cells express *slc32a1* (77 % of EGFP-positive V0v cells (30/39 cells counted in 2 embryos); Fig. 5f).

To determine whether V0v cells are becoming GABAergic and/or glycinergic we examined expression of markers of these two fates. We see no significant difference in the number of cells expressing GABAergic markers in single or double mutant embryos (Fig. 8m, n & r). In contrast, there is an increase in the number of cells expressing glycinergic markers. Interestingly, and in contrast to the *slc32a1* (*viaat*) result, we see a slight increase in both single mutants as well as a more pronounced increase in double mutants (Fig. 8i-l & q, Table 1). The increase in double mutants (approximately 28 cells) suggests that all V0v cells are becoming glycinergic.

### V0v cells become glycinergic through a novel Pax2-independent mechanism

All of the transcription factors that have been identified so far as specifying inhibitory spinal fates act through Pax2 [14–21]. In addition, Tlx1 and Tlx3, the only other transcription factors that have been identified as specifying excitatory spinal cord fates [4, 5, 8], work at least in part by down-regulating Pax2 [4]. Therefore, we decided to test if V0v cells turn on Pax2 expression in *evx1;evx2* double mutants. However, when we analyzed *pax2a* expression there was no significant difference in the number of cells expressing this gene in either the single or double mutants compared to WT embryos (Fig. 6b & e; Table 1). To further confirm this result, we performed immunohistochemistry using a Pax2 antibody that recognizes Pax2a and Pax2b [55]. Again, we saw no significant change in the number of cells expressing Pax2 protein in single or double mutants (Fig. 6d & f; Table 1). Finally, we also performed double-labeling experiments for Pax2 and EGFP in WT and mutant embryos that carried the *Tg(evx1:EGFP)*^*SU2*^ transgene, using either the Pax2 antibody or *in situ* hybridization with a mix of *pax2a*, *pax2b* and *pax8* probes. In each case we examined at least two WT embryos and two double homozygous mutants and we did not observe any double-labeled cells (Fig. 6h and data not shown).

Taken together these results show that V0v cells are becoming glycinergic through a Pax2-independent mechanism. This is the first time that a Pax2-independent mechanism of glycinergic specification has been identified in spinal cord neurons.

## Discussion

*Evx* genes are found in a wide range of animals ranging from corals to humans [72]. They encode transcription factors that contain both DNA-binding homeobox and C-terminal repressor domains [73–77]. Amniotes have two *Evx* genes (*Evx1* and *Evx2*) and teleosts, including zebrafish, have three (*evx1*, *evx2* and *eve1*), although only *evx1* and *evx2* are expressed in the spinal cord ([26, 32, 33, 59, 64]; this paper Figs. 2, 3, Additional file 1: Results and Figure S1). Interestingly, the genomic positions of these *evx* genes (adjacent to specific *Hox* clusters) along with phylogenetic analyses strongly suggest that the third *evx* gene in teleosts (*eve1*) is not the result of the extra genome duplication in the teleost lineage ([26, 78, 79], Additional file 1). Instead, it is likely that all three of these genes originated from the two rounds of whole genome duplication that occurred early in the vertebrate lineage [80] and *eve1* was later lost in the tetrapod lineage ([26, 78], Additional file 1).

Here we provide the first comprehensive analysis of the functions of Evx1 and Evx2 in spinal cord interneuron development in any vertebrate. We demonstrate that, within the spinal cord, both of these transcription factors are expressed exclusively by V0v cells. We also show that V0v cells are glutamatergic. These findings complement and extend those of Satou and colleagues [36], who reported that Evx2*-*expressing cells that develop from the *dbx1b* progenitor domain in zebrafish express the glutamateric maker *slc17a6b* and Talpalar and colleagues [9], who showed that mouse V0v cells express the glutamatergic marker *slc17a6* (*vglut2*). In addition, we confirm and extend previous reports that suggested that V0v neurons extend commissural axons [23, 27, 35, 36] and we identify these neurons as CoSA interneurons. Interestingly, while Satou and colleagues [36] observed similar V0v cell morphologies at the stages that we have examined, at later stages of development they also saw descending and bifurcating commissural excitatory V0 cells, suggesting that V0v cells may diversify morphologically at later developmental stages [36].

In mouse *Evx1* mutants, most V0v cells completely change their fate and acquire characteristics of V1 cells, the cell-type that normally forms ventral to V0v cells. Cells that would have formed V0v interneurons lose expression of Evx1 and Evx2 and instead express the V1 marker Engrailed1 (En1) and develop axon trajectories and migration patterns characteristic of V1 interneurons [23]. Most notably, their axon trajectories change from being contralateral to ipsilateral [23]. In addition, experiments in chick embryos revealed that ectopic Evx is sufficient to suppress Engrailed expression and therefore presumably V1 cell fate [23]. The role of Evx2 in mouse V0v cells is less well understood. Evx2 expression is dependent on Evx1, suggesting that it may be involved in the specification events described above [23], but spinal cord phenotypes of *Evx2* mutants have not been described in mouse and before this study, *Evx1;Evx2* double mutants had not been described in any vertebrate.

These amniote data suggest that Evx1 is required to inhibit the V1 fate in post-mitotic V0v cells. This global cell fate change is unusual for a transcription factor expressed in post-mitotic cells: it is more commonly seen with transcription factors expressed in spinal progenitor domains (e.g. [27–29, 81, 82]). For example, Nkx2.2 is a transcription factor expressed in the p3 progenitor domain and in *Nkx2.2* mutant mice, cells that would have formed V3 interneurons change their fates (transfate) and become motoneurons instead [83]. Similarly, Dbx1 is expressed in the progenitor domain (p0) from which V0 cells develop and in *Dbx1* mutant mice, cells that would have become V0v cells assume the characteristics of V1 cells [27, 28].

**Fig. 9:**
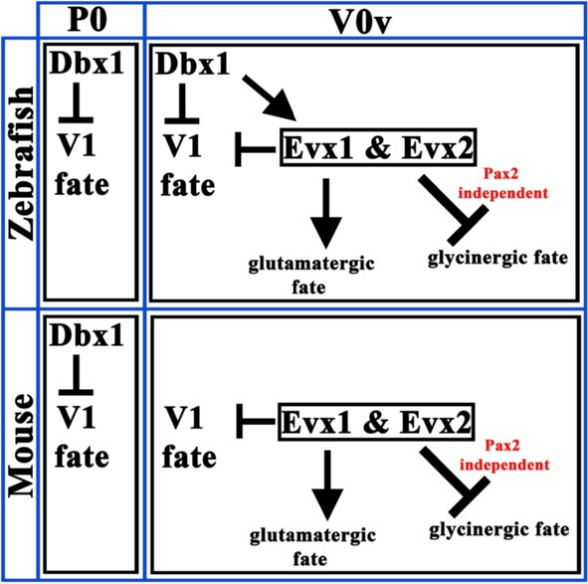
A Model for Evx Function in V0v cells. A possible model that would reconcile the different phenotypes of mouse and zebrafish *Evx1* mutants. In this model, Dbx1 expression in P0 cells is required for the expression of Evx1 and Evx2 in V0v cells (for simplicity this interaction is not shown) and Dbx1 can also independently repress the V1 fate. In zebrafish, Dbx1 expression persists for a while in newly formed V0v cells. This may be sufficient for the V1 fate to be inhibited in post-mitotic V0v cells, even in the absence of Evx1 and Evx2, thereby revealing other functions of Evx transcription factors. This could explain why V0v cells still form in *evx1;evx2* double mutants, but they express glycinergic rather than glutamatergic markers. In contrast, in mouse Dbx1 is expressed only in progenitor cells. Therefore, in newly formed V0v cells only Evx1 and Evx2 inhibit the V1 fate and in the absence of these transcription factors V0v cells transfate into V1 cells

Interestingly, our results are different and yield novel insights into Evx1 and Evx2 function in the spinal cord. We see no evidence of V0v cells transfating to V1 cells in zebrafish *evx1* and *evx2* single or double mutants. Notably, there is no increase in the number of cells expressing *eng1b*, which is specifically expressed in V1 cells, or Pax2, which is expressed by V1 and V0_D_ cells. Instead most V0v cells continue to express *evx1* mRNA and *Tg(evx1:EGFP)* and these EGFP-labelled V0v neurons have what appear to be normal CoSA axon morphologies. These data strongly suggest that V0v cells still form in zebrafish *evx1;evx2* double mutants and that they do not become a different class of neuron. One possible explanation for the differences between our results and the previously reported analyses in mouse might be evolutionary changes in the functions of Evx1 and Evx2. However, it is also possible that the consequences of removing these transcription factors are different in mouse and zebrafish because of variations in the expression of Dbx1 and/or the timing of V0v development. Interestingly, in this paper, we have shown that expression of *dbx1a* and *dbx1b* persists in at least some V0v cells in zebrafish. Therefore, it is possible that in zebrafish *evx1;evx2* mutants, Dbx might be able to inhibit post-mitotic V0v cells from becoming V1 cells (Fig. 9). Given the speed of zebrafish spinal cord development it is also possible that V0v cells become committed to their fate faster than in mouse and that, therefore, the window of time during which the V1 fate needs to be inhibited in V0v cells is much shorter in zebrafish than in mammals (see [84] for a different example of how the fast speed of zebrafish development can produce changes in spinal cord development). Regardless of how V0v global cell fate specification has evolved, the fact that V0v cells still form in zebrafish lacking Evx1 and Evx2, has provided us with a unique opportunity to identify Evx functions in V0v cells, independent of any role that these transcription factors may also have in repressing the V1 cell fate.

Our results show that in zebrafish, Evx1 and Evx2 act partially redundantly to specify the glutamatergic fate of V0v cells and inhibit an alternative glycinergic fate in these cells. Given that the only spinal cord cells that express *evx1* and *evx2* are V0v cells and that the only other trunk tissue that expresses either of these genes is the posterior gut, which expresses *evx1*, we consider that this requirement for Evx1 and Evx2 function is likely to be cell-autonomous. Interestingly, while there is a reduction of glutamatergic cells in both single and double mutants, expression of the inhibitory marker s*lc32a1* is only increased in double mutants, suggesting that the specification of glutamatergic fates and the inhibition of glycinergic fates may be independent processes which require different levels of Evx activity. However, in contrast to s*lc32a1*, the number of cells expressing the glycinergic marker *slc6a5* was slightly increased in single mutants, which suggests that the expression of different neurotransmitter transporter proteins is regulated independently and by distinct levels of Evx activity. These results are intriguing as they suggest that the regulation of neurotransmitter transporters and enzymes might be complex, with different components being regulated by distinct mechanisms.

V0v interneurons are a crucial part of locomotor circuitry as they are required for hindlimb left-right alternation during fast locomotion [9, 27, 34]. Therefore, changing the neurotransmitter fate of these cells might be expected to impair fast movements. Unfortunately, as *evx2* mutants die by larval stages, we were not able to assess whether *evx2* single mutants or *evx1;evx2* double mutants have locomotion defects. In addition, *evx1* single mutants lack joints in their fins [39], making it impossible to evaluate if any difference in *evx1* single mutant behavior is due to this fin phenotype or a locomotive defect. Interestingly, we did not observe any obvious changes in V0v cell morphology or axon trajectory in *evx1;evx2* double mutants. Given the changed neurotransmitter phenotype of V0v cells in these animals this might be considered surprising, although it is consistent with our previous analysis of V1 cells, that maintain their ipsilateral axon trajectories even when they lose their inhibitory fates in the absence of Pax2 and Pax8 [14]. It is still possible though that there are subtle changes in V0v cell wiring and/or changes in V0v cell connectivity in *evx1;evx2* double mutants as a result of their change in neurotransmitter fate. As there are fewer GFP-labelled V0v cells in *evx1;evx2* double mutants it is also possible that V0v cells with inappropriate neurotransmitter fates eventually die, although alternatively this reduction in the number of GFP-positive cells may just reflect autoregulation of Evx expression.

In this paper, we also describe the expression of a different transcription factor gene expressed by V0v cells, *skor2. Skor2* expression has also been reported in the mouse spinal cord but the cells that express it were not identified [85]. Our results show that *skor2* is expressed by a subset of V0v cells as well as at least one population of more dorsal excitatory spinal cord cells. We also demonstrate that expression of *skor2* in V0v cells requires Evx1 and Evx2 activity. Given that *skor2* is predominantly expressed by excitatory cells, it is possible that it acts downstream of Evx1 and Evx2 in V0v cells in either the specification of glutamatergic fates and/or the inhibition of glycinergic fates and that it might also have this function in other cells. However it is also possible that Skor2 acts downstream of Evx1 and Evx2 in some other as-yet-unidentified aspect of V0v cell specification. These alternatives can be tested by future loss-of-function analyses of Skor2.

Excitingly, in addition to demonstrating the roles of Evx1 and Evx2 in neurotransmitter specification, our data also show that these transcription factors function independently of Pax2 in specifying glutamatergic fates and inhibiting glycinergic fates. This is the first time that a Pax2-independent mechanism of inhibitory fate specification has been identified in the spinal cord. While several transcription factors have been identified that specify the inhibitory fates of particular spinal cord neurons, so far all of these act upstream of Pax2 [14–18]. In addition, as mentioned before, the only other transcription factors that have been identified as specifying excitatory spinal cord fates, Tlx1 and Tlx3 [4, 5, 8], work at least in part by down-regulating Pax2 [4]. Therefore, our finding that V0v cells become inhibitory in *evx1;evx2* double mutants but do not express Pax2 is a significant one as it demonstrates that there must be an additional Pax2-independent mechanism for specifying inhibitory neurons in the spinal cord.

## Conclusions

In conclusion, in this paper we demonstrate that zebrafish V0v cells express *evx1* and *evx2* and develop into excitatory (glutamatergic) CoSA interneurons. We also show that Evx1 and Evx2 are required, partially redundantly for expression of *skor2* and glutamatergic markers and inhibition of glycinergic markers in V0v cells and that in the absence of Evx1 and Evx2 function V0v cells become glycinergic through a novel Pax2-independent mechanism. Taken together, our data significantly increase our understanding of how neurotransmitter fates are specified and the genetic networks involved in these processes.

## Additional file

Additional file 1:
**Supplementary Data.** (PDF 2.67 mb)

## Abbreviations

CNE: conserved non-coding element
CNS: central nervous system
CoSA: commisural secondary ascending
DAB: 3,3’- diaminobenzidine
dpf: days post fertilization
FACS: fluorescent activated cell sorting
Gads: glutamic acid decarboxylases
h: hours post fertilization
LHS: left hand side
RHS: right hand side
WT: wild type
