# Supplementary material for: Evx1 and Evx2 specify excitatory neurotransmitter fates and suppress inhibitory fates through a Pax2-independent mechanism
Source: Neural Dev. 2016 Feb 19;11:5. doi: 10.1186/s13064-016-0059-9 (PMC4759709; doi:10.1186/s13064-016-0059-9)
Supplement: Additional file 1: — Supplementary Data. (PDF 2.67 mb) [file 13064_2016_59_MOESM1_ESM.pdf]

Additional File 1: Supplementary Data

Supplementary Methods

Phylogenetic and Synteny Analyses

To search for additional zebrafish Evx family members, we performed tblastn searches against the zebrafish genome using the NCBI website and zebrafish Eve1, Evx1 and Evx2 sequences. For these analyses, we used zebrafish Eve1, Evx1 and Evx2 protein sequences extracted from Ensembl. As searches using each of these three proteins generated almost identical results, tblastn analyses of additional genomes were performed with just one Evx family member. The human and mouse genomes were searched with zebrafish Eve1 to confirm that there is no Eve1 in mammals, and the medaka (*Oryzias latipes*), fugu (*Takifugu rubripes*), *Tetraodon nigroviridis* and stickleback (*Gasterosteus aculeatus*) genomes were searched with zebrafish Evx2, as this is the longest of the three zebrafish Evx proteins. In addition, the stickleback genome was searched with zebrafish Eve1 to further confirm that there is no Eve1 present in this genome. All of these tblastn searches were performed using the NCBI website with the exception of the *Tetraodon nigroviridis* genome, which was searched using the Ensembl website. For each genome, the location of each identified *Evx* gene was determined using Ensembl and visually inspected to identify if there was a syntenic relationship with a particular Hox cluster.

For phylogenetic analyses, protein sequences for Eve1, Evx1 and Evx2 were extracted from Ensembl (see Table S1 for genome assemblies and protein sequence identifiers). Where multiple peptides existed for a given protein, the longest peptide was chosen for alignment. These sequences were automatically aligned using the default parameters of the multi-sequence alignment program Clustal Omega (version 1.2.1) (http://www.ebi.ac.uk/Tools/msa/clustalo/) [1-3]. The resulting alignment was visually inspected and edited by eye [4]. The homeodomain region was identified in each case using Pfam http://pfam.xfam.org/ [5]. A conserved region of 71 amino acids, encompassing the homeodomain and flanking residues, was identified (Supplementary Data Figure 1A) and a phylogenetic tree generated using the neighbor-joining method (Supplementary Data Figure 1B) [1-3]. The phylogenetic tree was plotted using Phylodendron software (version 0.8d) (http://iubio.bio.indiana.edu/treeapp/treeprint-form.html).

Supplementary Results

Most *evx2*^{sa140} mutants die by larval stages and do not form swim bladders

As reported in the main paper, the *evx2*^{sa140} mutation does not appear to be homozygous viable. We do not observe any obvious morphological defects in mutant embryos during the first few days of development, but most *evx2* homozygous mutants die by larval stages. In addition, our data suggest that most *evx2* homozygous mutants do not develop a swim bladder. Incrosses from identified *evx2* heterozygous fish produce more embryos without swim bladders at 5 dpf than we would normally expect. To test whether this is because *evx2* mutants do not develop a swim bladder we genotyped all of the embryos from a clutch derived from such an incross. Of 20 embryos with swim bladders, 8 were homozygous WT, 11 were heterozygous and only one embryo was an *evx2* homozygous mutant. In contrast, 14 of the 24 embryos without a swim bladder were homozygous mutant, 5 were WT and 5 were heterozygous. This suggests that at least most *evx2* homozygous mutants do not develop a swim bladder. Given that lack of a swim bladder is often associated with embryonic lethal phenotypes in zebrafish [6], this may be at least one reason why *evx2* homozygous mutants do not survive. Consistent with this, when we split embryos from an incross of identified heterozygous *evx2* fish into those with a swim bladder (120) and those without a swim bladder (103), almost 90% of the embryos without a swim bladder (91/103) died by 10 dpf. Only one of the remaining embryos survived to one month and it died soon after. In contrast, 71% (85/120) of the embryos with a swim bladder survived beyond one month.

Teleosts have three *Evx* genes whereas mammals have two

Previous studies identified three *Evx* genes in zebrafish and two in tetrapods [e.g. 7, 8]. Given that the genomic sequence of many organisms has improved in recent years we decided to check whether any additional *Evx* genes could be identified in teleosts or mammals and also confirm the orthologous relationships between Evx proteins in different species. To that end we performed tblastn searches with Evx protein sequences against the genomes of zebrafish, fugu, *Tetraodon*, medaka, stickleback, mouse and human (see Supplementary Data Methods). In the two mammalian species we only identified Evx1 and Evx2. In all of the teleosts we only identified *evx1*, *evx2* and *eve1*, with the exception that no *eve1* gene was identified in stickleback, suggesting that this gene may have been lost in this species. Currently in the

zebrafish genome (version GRCz10) there are two annotated *evx1* genes. However, the genomic locations and coding sequences of these two genes are identical, the only difference is that one of the genes has two extra exons that contain 5' UTR sequences. Therefore, we are convinced that these annotations correspond to the same gene. Similarly in the current version of the fugu genome (4.0), there are two annotated *evx1* genes, (1 of 2) and (2 of 2). However, both of these genes have two transcripts and each of the transcripts is identical between the two genes. *evx1* (2 of 2) is located on scaffold_346 and is flanked by the *hoxaa* cluster, whereas *evx1* (1 of 2) is the only gene present on scaffold_3303, which is very short (8967 bp). Given that these two genes are identical in their sequence we are convinced that they correspond to the same gene and that scaffold_3303 corresponds to a subset of scaffold_346. This is also consistent with the fact that we did not identify any additional *evx1* genes in any of the other teleosts.

Previous analyses have suggested that *eve1* is not the result of the extra genome duplication in the teleost lineage [7-9], but that *eve1, evx1 and evx2* all originated from the two rounds of whole genome duplication that occurred early in the vertebrate lineage [10] and *eve1* was later lost in the tetrapod lineage [e.g. 7, 8, 11]. To further test this hypothesis and to confirm that the orthologous relationships of these genes are correctly identified in each of these species, we determined the syntenic relationships of all three *evx* genes with neighboring *hox* clusters. In all of the genomes that we examined, *eve1* is located adjacent to the *hoxba* cluster in teleosts and is absent in mammals, *evx1* is located next to the *hoxaa* cluster in teleosts and the *HoxA* cluster in mammals and *evx2* is located next to the *hoxda* cluster in teleosts and the *HoxD* cluster in mammals (Supplementary Data Figure 1D & E). We further confirmed the orthologous relationships between these different Evx family members using a phylogenetic analysis of a 71 amino acid conserved region encompassing the homeodomain (Supplementary Data Figure 1A and B).

Figure S1: Phylogenetic and Synteny Analyses of Evx Proteins

(A) Clustal analysis of Evx proteins using Clustal Omega (version 1.2.1). ***** = conserved amino acid, **:** = amino acid with strongly similar properties scoring >0.5 in the Gonnet PAM 250 matrix, **.** = amino acid with weakly similar properties scoring ≤0.5 in the Gonnet PAM 250 matrix. The region of each protein included in the alignment is indicated by numbers on either side of the sequence, which indicate amino acid positions in the full-length sequences. In each case the protein region corresponds to the homeodomain plus a few neighboring amino acids. (B) Phylogenetic tree of protein region aligned in (A) created using neighbor-joining method and plotted using Phylodendron software. (C) Key to species abbreviations used in A & B. (D & E) Schematic representations of genomic locations of *Evx* genes in mammals (D) and teleosts (E) relative to different *Hox* gene clusters. Note that not all 8 *hox* gene clusters are found in all teleosts. In addition, we found no evidence of an *eve1* gene in stickleback.

```
ory eve1    52     DQNRRHRTSF TREQLSRLEQ EYGRESYVSR PRRCELATAL NLPETTIKVW FQNRRMKDKR QRHSLPWPHP F    123
dan eve1    35     DQSRRHRTAF TREQLTRLEQ EYCKESYVSR PRRCELAAAL NLPETTIKVW FQNRRMKDKR QRHSLHWPNP L    105
fug eve1    36     DQSRRHRTAF TREQLSRLEQ EYVKESYVSR PRRCELATAL NLPETTIKVW FQNRRMKDKR QRHSLPWPHP L    106
tet eve1    43     DQSRRHRTAF TREQLSRLEQ EYVKESYVSR PRRCELATAL NLPETTIKVW FQNRRMKDKR QRHSLPWPHP L    113
dan evx1    152    DQMRRYRTAF TREQIARLEK EFYRENYVSR PRRCELAAAL NLPETTIKVW FQNRRMKDKR QRLAMTWPHP A    222
hom evx1    181    DQMRRYRTAF TREQIARLEK EFYRENYVSR PRRCELAAAL NLPETTIKVW FQNRRMKDKR QRLAMTWPHP A    251
mus evx1    181    DQMRRYRTAF TREQIARLEK EFYRENYVSR PRRCELAAAL NLPETTIKVW FQNRRMKDKR QRLAMTWPHP A    251
fug evx1    162    DQIRRYRTAF TREQIARLEK EFYRENYVSR PRRCELAAAL NLPETTIKVW FQNRRMKDKR QRLAMTWPHP A    232
tet evx1    153    DQIRRYRTAF TREQIARLEK EFYRENYVSR PRRCELAAAL NLPETTIKVW FQNRRMKDKR QRLAMTWPHP A    223
ory evx1    153    DQIRRYRTAF TREQIARLEK EFYRENYVSR PRRCELAAAL NLPETTIKVW FQNRRMKDKR QRLAMTWPHP A    223
gas evx1    149    DQIRRYRTAF TREQIARLEK EFYRENYVSR PRRCELAAAL NLPETTIKVW FQNRRMKDKR QRLAMTWPHP A    219
dan evx2    167    DQVRRYRTAF TREQIGRLEK EFYRENYVSR PRRCELAAAL NLPETTIKVW FQNRRMKDKR QRLAMSWPHP A    237
ory evx2    169    DQVRRYRTAF TREQIGRLEK EFYRENYVSR PRRCELAAAL NLPETTIKVW FQNRRMKDKR QRLAMSWPHP A    239
gas evx2    14     DQVRRYRTAF TREQIGRLEK EFYRENYVSR PRRCELAAAL NLPETTIKVW FQNRRMKDKR QRLAMSWPHP A    84
fug evx2    180    DQVRRYRTAF TREQIARLEK EFYRENYVSR PRRCELAAAL NLPETTIKVW FQNRRMKDKR QRLAMSWPHP A    250
tet evx2    161    DQVRRYRTAF TREQIARLEK EFYRENYVSR PRRCELAAAL NLPETTIKVW FQNRRMKDKR QRLAMSWPHP A    231
hom evx2    186    DQVRRYRTAF TREQIARLEK EFYRENYVSR PRRCELAAAL NLPETTIKVW FQNRRMKDKR QRLAMSWPHP A    256
mus evx2    189    DQVRRYRTAF TREQIARLEK EFYRENYVSR PRRCELAAAL NLPETTIKVW FQNRRMKDKR QRLAMSWPHP A    259
dme eve     68     PSVRRYRTAF TRDQLGRLEK EFYKENYVSR PRRCELAAQL NLPESTIKVW FQNRRMKDKR QRIAVAWPYA A    138
tri eve     139    QNIRRYRTAF TREQLARLEK EFFKENYVSR PRRCELAAQL NLPESTIKVW FQNRRMKDKR QRMAIAWPYA A    209
                    . **:**:* **:*:  ***: *: :*.**** *******: * ****:***** ********** ** :: **
```

A

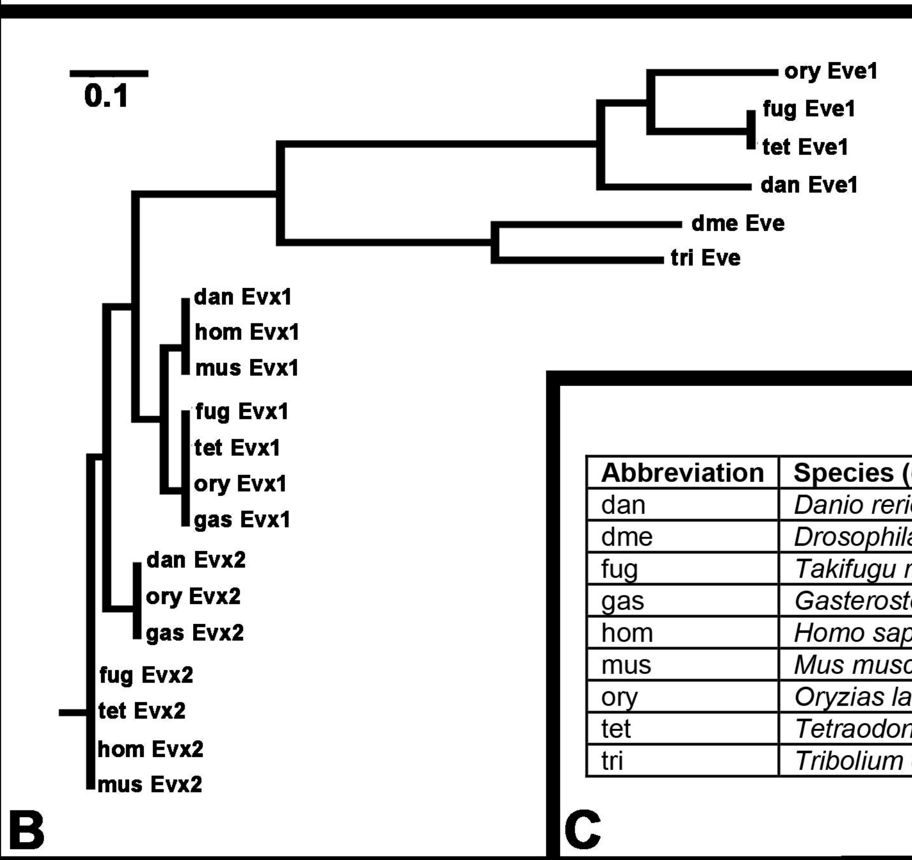

B

Abbreviation	Species (common name)
dan	*Danio rerio* (zebrafish)
dme	*Drosophila melanogaster*
fug	*Takifugu rubripes* (fugu)
gas	*Gasterosteus aculeatus* (stickleback)
hom	*Homo sapiens* (human)
mus	*Mus musculus* (mouse)
ory	*Oryzias latipes* (medaka)
tet	*Tetraodon nigroviridis*
tri	*Tribolium castaneum*

C

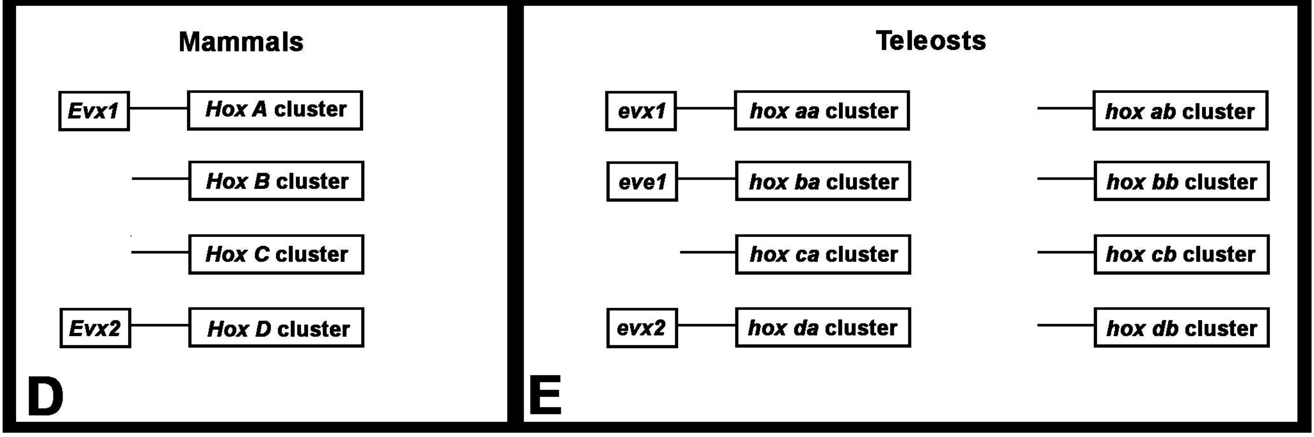

Figure S1

Table S1: Protein sequences used for phylogenetic analysis

Species	Protein Name	Ensembl Genome Assembly	Ensembl Protein Identifier	Protein Length (aa)	Homedomain Location (aa)
Drosophila melanogaster	Eve	BDGP6 (GCA_000001215.4)	FBpp0087478	376	71-127
Tribolium castaneum	TC-EVE	Tcas3	TC009469-PA	346	142-198
Danio rerio	Eve1	GRCz10 (GCA_000002035.3)	ENSDARP00000115133	225	38-94
Danio rerio	Evx1	GRCz10 (GCA_000002035.3)	ENSDARP00000141114	377	155-211
Danio rerio	Evx2	GRCz10 (GCA_000002035.3)	ENSDARP00000076765	418	170-226
Takifugu rubripes	Eve1	FUGU 4.0	ENSTRUP00000023584	244	39-95
Takifugu rubripes	Evx1	FUGU 4.0	ENSTRUP00000005213	384	165-221
Takifugu rubripes	Evx2	FUGU 4.0	ENSTRUP00000044950	431	183-239
Tetraodon nigroviridis	Eve1	TETRAODON 8.0	ENSTNIP00000004183	251	46-102
Tetraodon nigroviridis	Evx1	TETRAODON 8.0	ENSTNIP00000001316	375	156-212
Tetraodon nigroviridis	Evx2	TETRAODON 8.0	ENSTNIP00000004184	412	164-220
Oryzias latipes	Eve1	HdrR	ENSORLP00000021300	253	55-112
Oryzias latipes	Evx1	HdrR	ENSORLP00000006363	375	156-212
Oryzias latipes	Evx2	HdrR	ENSORLP00000021937	265	172-228
Gasterosteus aculeatus	Evx1	BROAD S1	ENSGACP00000009465	371	152-208
Gasterosteus aculeatus	Evx2	BROAD S1	ENSGACP00000006040	257	No assignment
Homo sapiens	EVX1	GRCh38.p5 (GCA_000001405.20)	ENSP00000419266	407	184-240
Homo sapiens	EVX2	GRCh38.p5 (GCA_000001405.20)	ENSP00000312385	476	189-245
Mus musculus	EVX1	GRCm38.p4 (GCA_000001635.6)	ENSMUSP00000031787	416	184-240
Mus musculus	EVX2	GRCm38.p4 (GCA_000001635.6)	ENSMUSP00000134131	475	192-248

Ensembl genome assemblies and protein sequence identifiers for proteins used in the phylogenetic analysis. Common names for the teleost species are as follows: *Danio rerio* (zebrafish), *Takifugu rubripes* (fugu), *Oryzias latipes* (medaka) and *Gasterosteus aculeatus* (stickleback). aa = amino acids. The homeodomain region was identified using Pfam http://pfam.xfam.org/.

Table S2: Gene Names and ZFIN identifiers

ZFIN identifiers (middle column) are provided for each of the genes used in this study along with common previous names. The column on the right indicates the references for the RNA probes used for *in situ* hybridization experiments.

Gene name	ZFIN ID	References for probe(s)
evx1	ZDB-GENE-980526-364	[12]
evx2	ZDB-GENE-980526-215	[13]
eve1	ZDB-GENE-980526-69	[14]
eng1b	ZDB-GENE-980526-6	[15]
pax2a (previously called *pax2.1*)	ZDB-GENE-990415-8	[16]
pax2b (previously called *pax2.2*)	ZDB-GENE-001030-4	[16]
pax8	ZDB-GENE-001030-3	[16]
dbx1a	ZDB-GENE-000128-8	[17]
dbx1b	ZDB-GENE-000128-11	[17]
*skor2 (*also called *zgc:153395*)	ZDB-GENE-060825-57	This paper
slc6a5 (previously called *glyt2*)	ZDB-GENE-050105-2	[18, 19]
gad2 (previously called *gad65*)	ZDB-GENE-030909-9	[18, 19]
gad1b (previously called *gad67*)	ZDB-GENE-030909-3	[18, 19]
slc17a6a (previously called *vglut2.2*)	ZDB-GENE-050105-4	[18, 19]
slc17a6b (previously called *vglut2.1*)	ZDB-GENE-030616-554	[18, 19]
slc32a1 (previously called *viaat*)	ZDB-GENE-061201-1	[20]

Supplementary Data References

1. Sievers F, Wilm A, Dineen D, Gibson TJ, Karplus K, Li W, Lopez R, McWilliam H, Remmert M, Soding J *et al*: Fast, scalable generation of high-quality protein multiple sequence alignments using Clustal Omega. *Molecular systems biology* 2011, 7:539.

2. McWilliam H, Li W, Uludag M, Squizzato S, Park YM, Buso N, Cowley AP, Lopez R: Analysis Tool Web Services from the EMBL-EBI. *Nucleic acids research* 2013, 41(Web Server issue):W597-600.

3. Goujon M, McWilliam H, Li W, Valentin F, Squizzato S, Paern J, Lopez R: A new bioinformatics analysis tools framework at EMBL-EBI. *Nucleic acids research* 2010, 38(Web Server issue):W695-699.

4. Thompson JD, Gibson TJ, Plewniak F, Jeanmougin F, Higgins DG: The CLUSTAL_X windows interface: flexible strategies for multiple sequence alignment aided by quality analysis tools. *Nucleic acids research* 1997, 25(24):4876-4882.

5. Finn RD, Coggill P, Eberhardt RY, Eddy SR, Mistry J, Mitchell AL, Potter SC, Punta M, Qureshi M, Sangrador-Vegas A *et al*: The Pfam protein families database: towards a more sustainable future. *Nucleic acids research* 2016, 44(D1):D279-285.

6. Yue MS, Peterson RE, Heideman W: Dioxin inhibition of swim bladder development in zebrafish: is it secondary to heart failure? *Aquatic toxicology* 2015, 162:10-17.

7. Amores A, Force A, Yan YL, Joly L, Amemiya C, Fritz A, Ho RK, Langeland J, Prince V, Wang YL *et al*: Zebrafish hox clusters and vertebrate genome evolution. *Science* 1998, 282(5394):1711-1714.

8. Avaron F, Thaeron-Antono C, Beck CW, Borday-Birraux V, Geraudie J, Casane D, Laurenti P: Comparison of even-skipped related gene expression pattern in vertebrates shows an association between expression domain loss and modification of selective constraints on sequences. *Evol Dev* 2003, 5(2):145-156.

9. Taylor JS, Braasch I, Frickey T, Meyer A, Van de Peer Y: Genome duplication, a trait shared by 22000 species of ray-finned fish. *Genome research* 2003, 13(3):382-390.

10. Holland PW, Garcia-Fernandez J, Williams NA, Sidow A: Gene duplications and the origins of vertebrate development. *Dev Suppl* 1994:125-133.

11. Minguillon C, Garcia-Fernandez J: Genesis and evolution of the Evx and Mox genes and the extended Hox and ParaHox gene clusters. *Genome biology* 2003, 4(2):R12.

12. Thaëron C, Avaron F, Casane D, Borday V, Thisse B, Thisse C, Boulekbache H, Laurenti P: Zebrafish evx1 is dynamically expressed during embryogenesis in subsets of interneurones, posterior gut and urogenital system. *Mechanisms of development* 2000, 99(1-2):167-172.

13. Sordino P, Duboule D, Kondo T: Zebrafish *Hoxa* and *Evx-2* genes: cloning, developmental expression and implications for the functional evolution of posterior *Hox* genes. *Mech Dev* 1996, 59:165 - 175.

14. Joly JS, Joly C, Schulte-Merker S, Boulekbache H, Condamine H: The ventral and posterior expression of the zebrafish homeobox gene eve1 is perturbed in dorsalized and mutant embryos. *Development* 1993, 119(4):1261-1275.

15. Batista MF, Lewis KE: Pax2/8 act redundantly to specify glycinergic and GABAergic fates of multiple spinal interneurons. *Dev Biol* 2008, 323(1):88-97.

16. Pfeffer PL, Gerster T, Lun K, Brand M, Busslinger M: Characterization of three novel members of the zebrafish *Pax2/5/8* family: dependency of *Pax5* and *Pax8* expression on the *Pax2.1* (*noi*) function. *Development* 1998, 125(16):3063-3074.

17. Gribble SL, Nikolaus OB, Dorsky RI: Regulation and function of Dbx genes in the zebrafish spinal cord. *Developmental dynamics : an official publication of the American Association of Anatomists* 2007, 236(12):3472-3483.

18. Higashijima S, Mandel G, Fetcho JR: Distribution of prospective glutamatergic, glycinergic, and GABAergic neurons in embryonic and larval zebrafish. *J Comp Neurol* 2004, 480(1):1-18.

19. Higashijima S, Schaefer M, Fetcho JR: Neurotransmitter properties of spinal interneurons in embryonic and larval zebrafish. *J Comp Neurol* 2004, 480(1):19-37.

20. Kimura Y, Okamura Y, Higashijima S: alx, a zebrafish homolog of Chx10, marks ipsilateral descending excitatory interneurons that participate in the regulation of spinal locomotor circuits. *J Neurosci* 2006, 26(21):5684-5697.
